# BDNF-Live-Exon-Visualization (BLEV) Allows Differential Detection of BDNF Transcripts *in vitro* and *in vivo*

**DOI:** 10.3389/fnmol.2018.00325

**Published:** 2018-09-27

**Authors:** Wibke Singer, Marie Manthey, Rama Panford-Walsh, Lucas Matt, Hyun-Soon Geisler, Eleonora Passeri, Gabriele Baj, Enrico Tongiorgi, Graciano Leal, Carlos B. Duarte, Ivan L. Salazar, Philipp Eckert, Karin Rohbock, Jing Hu, Jörg Strotmann, Peter Ruth, Ulrike Zimmermann, Lukas Rüttiger, Thomas Ott, Thomas Schimmang, Marlies Knipper

**Affiliations:** ^1^Department of Otolaryngology, Tübingen Hearing Research Centre (THRC), Molecular Physiology of Hearing, University of Tübingen, Tübingen, Germany; ^2^Department of Pharmacology, Institute of Pharmacy, Toxicology and Clinical Pharmacy, University of Tübingen, Tübingen, Germany; ^3^B.R.A.I.N. Centre for Neuroscience, Department of Life Sciences, University of Trieste, Trieste, Italy; ^4^Centre for Neuroscience and Cell Biology (CNC), Department of Life Sciences, University of Coimbra, Coimbra, Portugal; ^5^Centre for Integrative Neuroscience (CIN), University of Tübingen, Tübingen, Germany; ^6^Department of Physiology, Institute of Physiology, University of Hohenheim, Stuttgart, Germany; ^7^Transgenic Facility Tübingen, University of Tübingen, Tübingen, Germany; ^8^Instituto de Biologíay Genética Molecular, Universidad de Valladolid y Consejo Superior de Investigaciones Científicas, Valladolid, Spain

**Keywords:** BDNF detection, *Bdnf* exon-IV and -VI, transgenic BDNF reporter mice, activity-dependent BDNF expression, long-lasting plasticity changes

## Abstract

*Bdnf* exon-IV and exon-VI transcripts are driven by neuronal activity and are involved in pathologies related to sleep, fear or memory disorders. However, how their differential transcription translates activity changes into long-lasting network changes is elusive. Aiming to trace specifically the network controlled by exon-IV and -VI derived BDNF during activity-dependent plasticity changes, we generated a transgenic reporter mouse for ***B****DNF*-***l****ive-****e****xon*-***v****isualization (BLEV*), in which expression of *Bdnf* exon-IV and -VI can be visualized by co-expression of CFP and YFP. CFP and YFP expression was differentially activated and targeted in cell lines, primary cultures and BLEV reporter mice without interfering with BDNF protein synthesis. CFP and YFP expression, moreover, overlapped with BDNF protein expression in defined hippocampal neuronal, glial and vascular locations *in vivo*. So far, activity-dependent BDNF cannot be explicitly monitored independent of basal BDNF levels. The BLEV reporter mouse therefore provides a new model, which can be used to test whether stimulus-induced activity-dependent changes in BDNF expression are instrumental for long-lasting plasticity modifications.

## Introduction

Brain derived neurotrophic factor (BDNF), identified and purified in 1982 (Barde et al., 1982), is a key modulator of synaptic function during homeostatic readjustment processes and a master regulator of energy homeostasis (for review see: Bramham and Messaoudi, 2005; Rauskolb et al., 2010). Despite its importance, the influence of BDNF on circuit stabilization in the adult system, or its complex role in numerous brain and cardio-vascular diseases (Kuipers and Bramham, 2006; Marosi and Mattson, 2014; Leal et al., 2017), is still not completely understood (Nahmani and Turrigiano, 2014). Several factors impede detailed analysis. On the one hand, expression of BDNF in the mature CNS is extremely low and not restricted to neurons (Danzer and McNamara, 2004; Dieni et al., 2012), but also found in platelets (Chacón-Fernández et al., 2016), capillary endothelial cells (Donovan et al., 2000), microglia, and astrocytes (Ferrini and De Koninck, 2013; Parkhurst et al., 2013). Most enigmatic, however, is the complex structure of the BDNF gene, which consists of eight non-coding exons (I-VIII), which are alternatively spliced to the protein-encoding exon-IX. Transcription of each of the resulting mRNAs is regulated differently in terms of temporal and spatial location, additionally some transcripts show stimulus- and activity-dependence (Pattabiraman et al., 2005; Chiaruttini et al., 2008). The resulting transcripts in turn display different stability, targeting, and translatability (Timmusk et al., 1993; West et al., 2014). Ultimately, each transcript is translated into an identical BDNF peptide, cleaved and released as mature BDNF (Yang et al., 2009). BDNF transcripts containing *Bdnf* exon-IV and -VI are particularly interesting as their translation is directly or indirectly regulated by changes in neuronal activity (Hong et al., 2008; West et al., 2014; Tuvikene et al., 2016) and their dysregulation is linked to various brain pathologies related to sleep, loss of fear memory (Hill et al., 2016), and depression (Marosi and Mattson, 2014). BDNF-TrkB receptor signaling is crucial for activity-dependent regulation of synaptic strength in various brain regions (Kellner et al., 2014). Moreover, activity-dependent regulation of synaptic strength was previously suggested to play a role during long-lasting adaptation of brain responses to external demand. Accordingly, only the coincidence of for example glucocorticoid function acting on mitochondria and dendritic spines together with context-specific activity (e.g., motor learning), lead to long-lasting spine formation, memory consolidation and behavioral performance (see for a review: Jeanneteau and Arango-Lievano, 2016). In this context the potential function of activity-dependent BDNF to provide context information cannot be tested due to difficulties in its detection in the adult organ (Dieni et al., 2012), and unfeasibility to extract activity-dependent BDNF from background BDNF levels.

To investigate whether activity-dependent *Bdnf* exon-IV or -VI promoter usage provides context-specific information during task-specific learning, we generated a knock-in reporter mouse line for ***B****DNF*-***l****ive-****e****xon-****v****isualization* (BLEV). In contrast to previous studies analyzing the distinct functions of *Bdnf* transcripts through deletion of promoter function (Hong et al., 2008; Sakata et al., 2010; Parkhurst et al., 2013; Mallei et al., 2015), we generated a BDNF knock-in reporter mouse. In the BLEV reporter mouse line, the marker proteins CFP and YFP (cyan- and yellow-fluorescent protein) tag the sites, where mRNA containing the activity-dependent *Bdnf* exon-IV or exon-VI is translated. This allows monitoring of exon-IV and exon-VI promoter usage *in vitro* and *in vivo* above the background of basal BDNF levels. We verify that the knock-in does not interfere with the normal BDNF transcription, translation or protein function and approve the specific detection of activity-driven BDNF transcript changes in the brain.

The BLEV reporter mouse thus constitutes the first model to allow selective and sensitive tracing of activity-dependent *Bdnf* transcripts in functional neuronal networks *in vitro* and *in vivo*, without impairing normal BDNF protein functions.

## Materials and methods

### Animals

Animal care and use and experimental protocols correspond to national and institutional guidelines and were reviewed and approved by the animal welfare commissioner and the regional board for animal experimentation. All experiments were performed according to the European Union Directive 2010/63/EU for the protection of animals used for experimental and other scientific purposes.

### Vector construct for a transgenic BDNF mouse

The *Bdnf* exon-IV sequence is extended by CFP and the *Bdnf* exon-VI sequence by YFP, both containing a stop codon. The translation of *Bdnf* exon-IX is enabled by an IRES sequence, which keeps the mRNA at the ribosome, despite the presence of a stop codon. Additionally, the growth-associated protein 43 (GAP43), is added to anchor the fluorescent proteins at the site of translation. This allows differential monitoring of the non-coding *Bdnf* exon-IV and *Bdnf* exon-VI by the fluorescent proteins CFP and YFP without interfering with *Bdnf* exon-IX.

**Figure 1:**
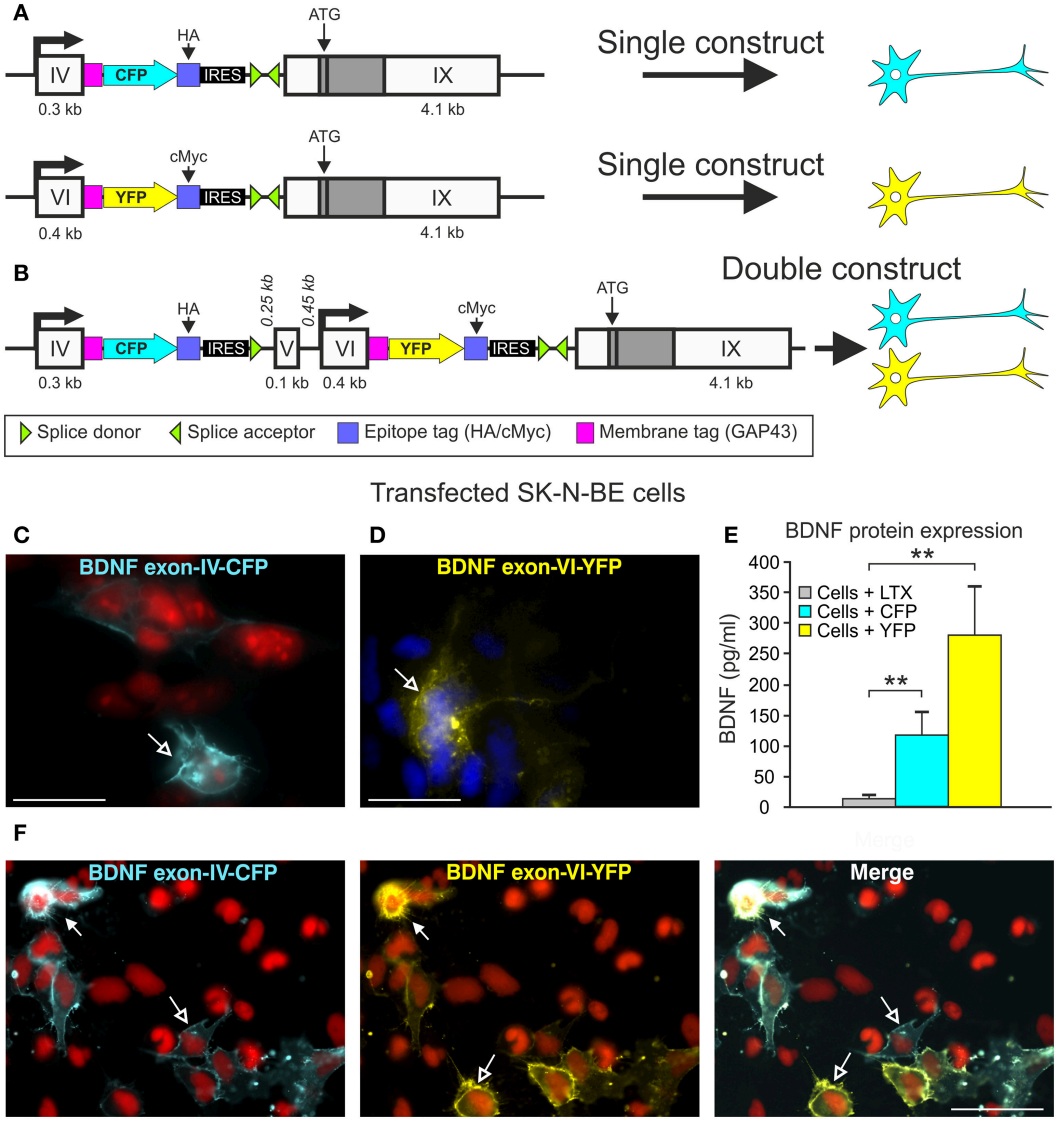
BLEV construct and verification of the in *vitro*. **(A)** Schematic drawing of the *Bdnf* exon-IV-CFP and *Bdnf* exon-VI-YFP single constructs used in cell culture. **(B)** Schematic drawing of the *Bdnf* exon-IV-CFP and exon-VI-YFP double construct used in cell culture. **(C,D)** CFP expression of the *Bdnf* exon-IV-CFP single construct (blue, left panel, nuclear staining: propidium iodide, in red) and YFP expression of the *Bdnf* exon-VI-YFP single construct (yellow, right panel, nuclear staining: DAPI, in blue). **(E)** ELISA of BDNF protein in SK-N-BE cells transfected with either the *Bdnf* exon-IV-CFP single construct or the *Bdnf* exon-VI-YFP single construct. BDNF is not expressed in cells treated with Lipofectamine (LTX, gray bar) only. BDNF is expressed in SK-N-BE cells transfected with either of the two single constructs (blue bar: *Bdnf* exon-IV-CFP; yellow bar: *Bdnf* exon-VI-YFP). Data are shown as mean BDNF concentration (pg/ml) ± SEM [1-way ANOVA: *F*_(3, 36)_ = 8.35 *p* = 0.0002; post-test 2-sided Student's *t*-test: CFP *p* = 0.008; YFP *p* = 0.003; done in duplicate for 4 independent experiments]. **(F)** SK-N-BE cells transfected with the *Bdnf* exon-IV-CFP exon-VI-YFP double construct. The left panel shows the CFP expression (blue), the middle panel shows the YFP expression (yellow), and the right panel shows a merged image of *Bdnf* exon-IV-CFP and *Bdnf* exon-VI-YFP expression. Open arrows show cells expressing either CFP or YFP, filled arrows show cells expressing CFP and YFP. Nuclear staining in red (propidium iodide). **(C,D)** scale bars = 20 μm; **(F)** scale bar = 50 μm.

In detail, to generate a mouse line in which different *Bdnf* exons are labeled by different fluorescent markers (Figure 1A), *Bdnf* exon-IV and -VI were amplified from genomic DNA. Primers were designed to amplify *Bdnf* exon-IV (5′-TAGAACCTTGGGGACCATGCTGTGCTGTATGAG-3′, 5′-CAGCACAGCATGGTCCCCAAGGTTCTAGACTC-3′; 5′-GAGAAAGCGCAGGGACCATGCTGTGCTGTGCTGTATGAG-3′; 5′-CAGCACAGCATGGTCCGTGCGCTTTCTCTGCTGCC-3′) or *Bdnf* exon-VI (5′-GTGGGGCAAAGCGAACTGTGA-3′, 5′-CGCAACCCCCATAACGACCAG-3′) including their promoter regions. The PCR product was digested with the enzymes XhoI, SbfI, and MluI for exon-IV and XhoI, AccIII, and MluI for exon-VI to separate the two exons. In a next step, oligonucleotides specific for the membrane anchoring sequence of GAP43, the hemagglutinin (HA) epitope tag, and cMyc (cellular myelocytomatosis oncogene) epitope tag were designed. GAP43 was cloned into the 5′-multiple cloning site (MCS) of the CFP and the YFP vectors (Takara Bio Europe/Clontech; https://www.takarabio.com/assets/documents/Vector%20Documents/pAmCyan%20Vector%20Information.pdf, https://www.takarabio.com/assets/documents/Vector%20Documents/PT3481-5_080612.pdf). The HA tag was inserted into the 3′-MCS of the CFP vector, and the cMyc tag was inserted into the 3′-MCS of the YFP vector. A chimera of *Bdnf* exon-IV and the CFP vector containing GAP43 and HA was amplified. The same was done for *Bdnf* exon-VI and the YFP vector containing GAP43 and cMyc. To make use of an internal ribosomal entry site (IRES), the two PCR products were cloned into a pIRES vector (Takara Bio Europe/Clontech, http://www.takara.co.kr/file/manual/pdf/PT3266-5.pdf). The PCR product containing *Bdnf* exon-IV, GAP43, CFP, and HA was inserted into the MCS A of the pIRES vector. The same was done for the PCR product containing *Bdnf* exon-VI, GAP43, YFP, and cMyc. To design a construct containing *Bdnf* exon-IV and -VI in one vector, *Bdnf* exon-VI, GAP43, YFP, cMyc, and pIRES were cut out of one pIRES vector and cloned into the MCS B of the pIRES vector, which already contained *Bdnf* exon-IV, GAP43, CFP, and HA. From this pIRES vector, the part containing *Bdnf* exon-IV, GAP43, CFP, HA, pIRES and *Bdnf* exon-VI, GAP43, YFP, cMyc, pIRES were inserted together with the loxP sites of the pMCS 5 vector (http://www.mobitec.com/cms/products/bio/04_vector_sys/multiple_cloning_site_pmcs5.html, kindly provided by Prof. Dusan Bartsch; Central Institute of Mental Health, Department of Molecular Biology, Mannheim, Germany), into the pEasyFloxII/SK62 vector between the neomycin cassette and the HSV-TK cassette. The 5′-homologous sequence was also inserted into the pEasyFloxII/SK62 vector in front of the neomycin cassette. This vector construct was used to generate the transgenic mouse line.

### Generation of the transgenic BDNF mouse

The construct was linearized with NotI and electroporated into HM1 embryonic stem (ES) cells (Magin et al., 1992). ES cell clones were selected with neomycin. Recombinant ES cell clones were verified by PCR using oligonucleotides for the 5′-end (for: 5′-GAGTTGGGAGAATATTAGGC-3′; rev: AGGTAGCCGGATCAAGCGTATGCAGC-3′) and the 3′-end (for: 5′-GTCCTGCTGGATATATACATGGGGCAG-3′; rev: 5′-GTCAACTTATAATTACCGGTTCC-3′) resulting in PCR products of either 2.5 or 6.7 kb, respectively. Positive clones were screened additionally by Southern blot. In brief, isolated DNA was digested using EcoRV and SalI (Roche). DNA fragments were separated on a 0.8% agarose gel. The DNA was blotted onto a nylon membrane and fixed. Different DNA probes were hybridized to the membrane at 68°C overnight. On the next day, bands were visualized using the detection starter Kit II (Roche). The probes were specific either for the 5′-end, the 3′-end and two internal sequences, covering either parts of the transgenic exon-IV or -VI. Due to the restriction sites, the WT and the transgenic allele could be differentiated. For the 5′-end probe, the WT band was 9.8 kb, the band of the transgenic allele was 3 kb; for the 3′-end, the WT and the transgenic band was 12.7 kb. For the probe covering exon-IV, a WT band of 9.8 and 5.3 kb band for the transgenic allele were obtained. The probe covering exon-VI resulted in a 9.8 kb band for the WT and a 6.3 kb band for the transgenic allele (Figure 3C, Supplementary Figure 1). A selected ES cell clone was used for injection into blastocysts. Chimeras were carried out by foster mothers. Deletion of the Neo cassette was achieved by breeding the offspring with Pgk-*Cre* mice (Lallemand et al., 1998). Genotyping of these mice was performed using oligonucleotides for *Cre* (for: 5′-ACGACCAAGTGACAGCAATA-3′, rev: 5′-CCATGCCTCGACCAGTTTAG-3′). For genotyping the transgenic BDNF mouse, two primer sets were used. The insert PCR of CFP (for: 5′-GAACAGGAGTACATATCGGCC-3′, rev: 5′-TTCATATGACATTCCGTCAGG-3′) resulted in a 437 bp PCR product for the transgenic mouse and no PCR product for the WT. To verify the different genotypes, additional PCRs were performed using the forward primer 5′-GAACAGGAGTACATATCGGCC-3′ and either the reverse primer 5′-GAACACACAATGAAACTACACAGAG-3′ for the WT resulting in a 428 bp product, or 5′-TTCATATGACATTCCGTCAGG-3′ for the transgene (437 bp). The BLEV mouse line (B6;129-Bdnftm1(ex4CFPex6YFP)MknixB6;HM1-Pgktm1(Cre)Lni/Mkni) was maintained by breeding heterozygotes.

## Cell culture

### Vector construct for cell culture

For the constructs used in cell culture, *Bdnf* exon-IX was inserted in the MCS B of the pIRES vectors containing either *Bdnf* exon-IV, GAP43, CFP, and HA, or *Bdnf* exon-VI, GAP43, YFP, and cMyc (single constructs, Figure 1A). To receive the double construct, *Bdnf* exon-IX was cut out of the pIRES vector containing *Bdnf* exon-IV, GAP43, CFP, and HA. Instead, the vector part *Bdnf* exon-VI, GAP43, YFP, cMyc, and *Bdnf* exon-IX from the single construct was inserted into the MCS B (Figure 1B).

### Cell culture and transfection

SK-N-BE cells, kindly provided by PD Dr. Ulrike Naumann (Hertie Institute, Tübingen, Germany), were grown on 75 cm^2^ flasks in 11 ml Dulbecco's Modified Eagle's Medium (DMEM) (Gibco/Life Technologies) containing 10% fetal calf serum (Gibco/Life Technologies) and 1% penicillin and streptomycin (Gibco/Life Technologies). Cells were sub-cultured for 2 days at 3 × 10^4^ cells/well (BD Bioscience), ensuring cells would be in the log phase of differentiation. The cells were transfected with two different BDNF single constructs or the double construct (Figures 1A–D) when they were 50–80% confluent using the lipid-based transfection reagent LTX (Lipofectamine, Life Technologies) according to manufacturer's guidelines. For each DNA construct, 3.5 μg of DNA was gently diluted in 700 μl of DMEM together with 3.5 μl of plus reagent and 7 μl of LTX and incubated for 25-30 min at room temperature. DNA/LTX solution (about 700 μl) and 1,300 μl of serum-free medium were added to each well and mixed by gently rocking the plate for 5 h. The culture medium was changed to standard medium and the cells were incubated at 37°C, 5% CO_2_ for 24 h.

### ELISA (enzyme-linked immunosorbent assay) for BDNF

For SK-N-BE cells transfected with 1.5 μg of either single construct (Figure 1A) or treated with Lipofectamine only, ELISA for BDNF protein was performed using the ELISA kit from Chemicon. BDNF content was determined from A_450_ readings of human BDNF standards.

### Primary neuronal culture

Primary mixed neuronal cultures of cortical neurons (mice postnatal day P1-7) or hippocampus (rat, embryonic day E17-18) were prepared following the procedure described by Goslin et al. (1998) and Salazar et al. (2017).

For transfection, primary cortical neurons were plated 7–11 days and transfected with 1–2 μg of the double construct *Bdnf* exon-IV-(HA)-CFP/*Bdnf* exon-VI-(cMyc)-YFP-exon-IX and 1–2 μl Lipofectamine 2000 (Life Technologies) solution (1 mg/ml) diluted in 50–80 μl MEM without serum and antibiotics. The Lipofectamine 2000 and DNA mix was removed 1 h after transfection. The cells were returned to the initial conditioned medium and were incubated in a 5% CO_2_-humidified incubator at 37°C for 7 d to allow the expression of transfected constructs.

### Stimulation of hippocampal primary culture with bicuculline

Hippocampal neurons transfected at 7 DIV were stimulated at 15 DIV with 50 μM bicuculline, 2.5 mM 4-Aminopyridine and 10 μM glycine for 3 h as previously described (Costa et al., 2016). They were fixed for 15 min at RT in 4% paraformaldehyde in PBS and mounted in fluorescence mounting medium (DAKO). Fluorescence was analyzed with a Nikon C1Si confocal microscope (Nikon Instruments Europe BV, Amsterdam, Netherlands). A series of optical images at 0.2 μm increments along the “z” axis of the cells stained was acquired. Images were processed for z-projection and for illustration purposes by using ImageJ (NIH, Bethesda, USA) and Adobe Photoshop CS4 (Adobe Systems, San Jose, CA). For immunohistochemistry (Supplementary Figure 2) hippocampal neurons were fixed as described above. They were permeabilized with 0.3% Triton X-100 in PBS and incubated with 10% BSA in PBS, for 30 min at 37°C, to block non-specific staining. Afterwards they were incubated for 2 h at 37°C with the primary antibodies against HA (-CFP) and cMyc (-YFP) diluted in 3% BSA in PBS, for antibody information see Supplementary Table 1. The cells were washed 6 times with PBS for 2 min and incubated with the secondary antibodies (see Supplementary Table 1), for 45 min at 37°C. Afterwards the coverslips were mounted with a fluorescence mounting medium (DAKO). Here fluorescence images were acquired using a Carl Zeiss LSM 710 confocal microscope with a Plan-Apochromat 63 × /1.4 objective using identical settings, with the following excitation lasers/wavelengths: DPSS 561-10/561nm [Red; to visualize *Bdnf* exon-IV-(HA)-CFP], and HeNe633/633nm [Far-Red; to visualize *Bdnf* exon-VI-(cMyc)-YFP].

### Kainic acid injection

Two to three-month-old homozygous BLEV mice of either sex were injected intraperitoneally with 12 mg/kg kainic acid (Tocris) (KA). This concentration has been previously shown to induce an activity-dependent expression of *Bdnf* exon-IV and -VI in the hippocampus (Chiaruttini et al., 2008). Control animals received the same amount of 0.9% NaCl solution (Fresenius) (vehicle-treated animals). Two hours after injection animals were sacrificed. Animals developed hardly any seizures as they were generated on a C57BL/6N background, which was shown to be resistant to KA-induced insults (Mclin and Steward, 2006).

### Hearing measurements

The hearing function of 2–3 months old homozygous BLEV mice of both sexes was studied by measuring auditory brainstem responses (ABRs), as described previously (Zuccotti et al., 2012; Rüttiger et al., 2013).

### Tissue preparation

For RNA and protein isolation, brains were dissected with small forceps and immediately frozen in liquid nitrogen and stored at −80°C before use. Brain and cochlear tissue for immunohistochemistry was prepared as previously described (Singer et al., 2016).

### RNA isolation

RNA was extracted from brain tissues with a ready-to-use kit according to the manufacturer's protocol (Macherey-Nagel).

### Semi-quantitative reverse transcription and polymerase chain reaction (RT-PCR)

Transcription of RNA to cDNA was carried out as previously described (Tan et al., 2007). Transcribed cDNA was amplified using PuReTaq Ready-To-Go PCR beads (Amersham Biosciences). Specific forward- and reverse primers for *Bdnf* exon-IX (for: 5′-GAAGCAAACGTCCACGGACAA-3′, rev: 5′-CTGGATGAGGACCAGAAGGTT-3′, 171 bp) were used. Glyceraldehyde 3-phosphate dehydrogenase (GAPDH, for: 5'-TCTACTGGTGTCTTCACCACCA-3′, rev: 5′-ACTGAGGACCAGGTTGTCTCCT-3′, 600 bp) was used as housekeeping gene. Primers for *Bdnf* exons I, II, III, IV, V, VI, VII, VIII and XIA were used according to (Aid et al., 2007). A probe containing the same reagents except the cDNA was used as a negative control. The resulting PCR products were separated on 1.5% agarose gels by electrophoresis and stained with ethidium bromide.

### Protein isolation and western blot

For isolation of cMyc-tagged proteins and HA-tagged proteins, the Mild Purification kit and the HA-tagged Protein Purification kit were used, respectively (BiozolDiagnostica). In brief, tissues were dissolved in a lysis buffer (CelLytic M, Sigma-Aldrich) and incubated for 1 h with anti-cMyc or anti-HA tag beads suspension. The suspension was then centrifuged and washed; cMyc- and HA-tagged proteins were eluted with Elution Peptide Solution from the kit.

For BDNF Western blot, co-immunoprecipitation was performed using the Catch and Release v2.0 (Merck Millipore). In brief, tissues were dissolved in lysis buffer (CelLytic M). After preparing the columns with Catch and Release wash buffer, tissue lysate, antibody (anti-BDNF, Genaxxon Bioscience), antibody capture affinity ligand and wash buffer were added to the column. Loaded columns were incubated overnight at a mixer at room temperature. On the next day, the column was centrifuged and washed followed by the elution of the proteins.

Proteins were separated by electrophoresis and placed on a transfer membrane; non-specific epitopes of the membrane were blocked with 5% milk powder solution and incubated overnight at 4°C with the primary antibody (see Supplementary Table 1). On the second day, the membrane was washed three times with Tris buffer/0.1% Tween 20; the secondary antibody (HRP-linked ECL anti-rabbit IgG or HRP-linked ECL anti-mouse IgG; GE Healthcare) was incubated for 1 h at room temperature in a sealed envelope. The membrane was washed again three times with Tris buffer/0.1% Tween 20. Finally, the protein bands were visualized with ECL Prime WB Detection Reagent (GE Healthcare) using the Proxima 2700 (Isogen Life Science).

### Immunohistochemistry

Brain tissue were isolated, fixed, sectioned, and stained as previously described (Tan et al., 2007; Singer et al., 2016). For antibody information see Supplementary Table 1.

## Data analyses

### Statistics

All Statistical results and information can be found in the figure legends and in Supplementary Table 2. In figures, significance is indicated by asterisks (^*^p < 0.05, ^**^p < 0.01). For animal experiments Power analyses is performed a priori for the applications for animal experiments. A sample size of 4-5 animals per group is sufficient to evaluate a difference in hearing threshold of 10 – 15 dB SPL (alpha 0.05, Power 0.8). For molecular analyses a sample size of 3 – 4 is sufficient to evaluate a difference in gene/protein expression of 15% (alpha 0.05, Power 0.8). For analyses of hearing thresholds no data were excluded, in all mice a hearing threshold could be measured. Molecular samples were excluded when the standard curves were not fitting (ELISA), bands were missing or the housekeeping genes in PCR or Western Blot were irregularly expressed.

### ELISA

Data are shown as mean BDNF protein concentration in (pg/ml) (± SEM). Data were statistically analyzed by Student's *t*-test with α = 0.05.

### PCR

The intensity of the bands was analyzed using the TotalLab Quant software (TotalLab Ltd.). Band intensities of BDNF were normalized to housekeeping gene GAPDH. Results are depicted in relation to *Bdnf* exon-IX expression of WT mice, which was set to 1 (dotted line) as mean ± % SEM. Data were analyzed by 1-way ANOVA with α = 0.05, post-test: Bonferroni-Holms (GraphPad Prism). For the untranslated *Bdnf* exons I, II, III, IV, VI, and XIA mean expression values ± SEM are shown for WT and homozygous mice. Data were analyzed for each exon by 2-sided Student's *t*-test with α = 0.05 (GraphPad Prism). For original picture see Supplementary Figure 6D.

### Western blot

The intensity of the bands was analyzed using the TotalLab Quant software. Band intensities of the genes of interest were normalized to housekeeping gene GAPDH. For BDNF results are depicted in relation to BDNF expression of WT mice, which was set to 1 (dotted line) as mean ± % SEM. Data were analyzed by 1-way ANOVA with α = 0.05, post-test: Bonferroni-Holms. For tissue from kainic acid-treated mice, results are shown as % of vehicle-treated mice as mean ± % SEM. Data were analyzed by a 1-sided Student's *t*-test with α = 0.05 (GraphPad Prism). For original Blots see Supplementary Figures 6B,C.

### Hearing measurements

Click-ABR measurements were analyzed by 1-way ANOVA with α = 0.05, post-test: Bonferroni-Holms. Frequency-ABR measurements were group analyzed by multiple *t*-test with α = 0.05, corrected for multiple comparison using the Holm-Sidak method (GraphPad Prism). Data are shown as mean ± SD.

### Fluorescence analysis of brain immunohistochemistry

Sections shown here were viewed using an Olympus BX61 microscope (Olympus, Center Valley, PA, USA) equipped with an X-Cite Lamp (Olympus). Images were acquired using an Olympus XM10 CCD monochrome camera and analyzed with cellSens Dimension software (OSIS).

To increase spatial resolution, slices were imaged over a distance of 13–15 μm in steps of 0.49 μm within an image-stack along the *z*-axis (*z*-stack) followed by 3-dimensional deconvolution, using a cellSens Dimension built-in algorithm. Typically *z*-stacks consisted of 27–30 layers, for each layer one image was acquired per fluorochrome.

Picture acquired from brain section stained for parvalbumin (PV), were analyzed using the free software ImageJ. (NIH, Bethesda, MD, USA). For each section, three pictures for each single channel (YFP, CFP, PV) were saved and analyzed independently.

For integrated density analysis following vehicle and kainic acid treatment, images from equivalent CA3 regions between treatments were analyzed with ImageJ software to quantify integrated density of CFP and YFP staining within each image for each replicate. Threshold adjustments were set to ensure quantification of only positive immunostaining.

### Data availability

The datasets generated during and/or analyzed during the current study are available from the corresponding author upon request.

## Results

### Generation of a transgene to monitor transcript IV- and transcript VI-specific BDNF synthesis

In order to generate a system to monitor the sites of transcript-specific BDNF synthesis subsequent to activity-dependent activation of exon-IV or exon-VI promoters *in vivo*, we generated constructs, which allow ***B****DNF-****l****ive-****e****xon-****v****isualization* (BLEV) (Figure 1). In the BLEV constructs *Bdnf* exons-IV and -VI are labeled by two different fluorescence proteins: the *Bdnf* exon-IV sequence is extended by CFP and the *Bdnf* exon-VI sequence by YFP, both containing a stop codon. To retain translation of the coding *Bdnf* exon-IX, we introduced an IRES (internal ribosome entry site) sequence, which keeps the mRNA at the ribosome, despite the presence of a stop codon within CFP or YFP. Additionally, the fluorescent proteins were fused to the membrane tag GAP43, in order to anchor them at the site of translation. This design allows localization of when and where *Bdnf* exon-IV and -VI mRNA is used by CFP and YFP fluorescence without interrupting post-translational processing of BDNF (Figures 1A,B). Two different epitope tags (HA, cMyc) were incorporated into the construct to facilitate the quantification of CFP or YFP expression by e.g., Western blot (Figures 1A,B). Thus, the design of these constructs allows for the quantification of the amount of transcript specific mRNA used for protein translation.

**Figure 2:**
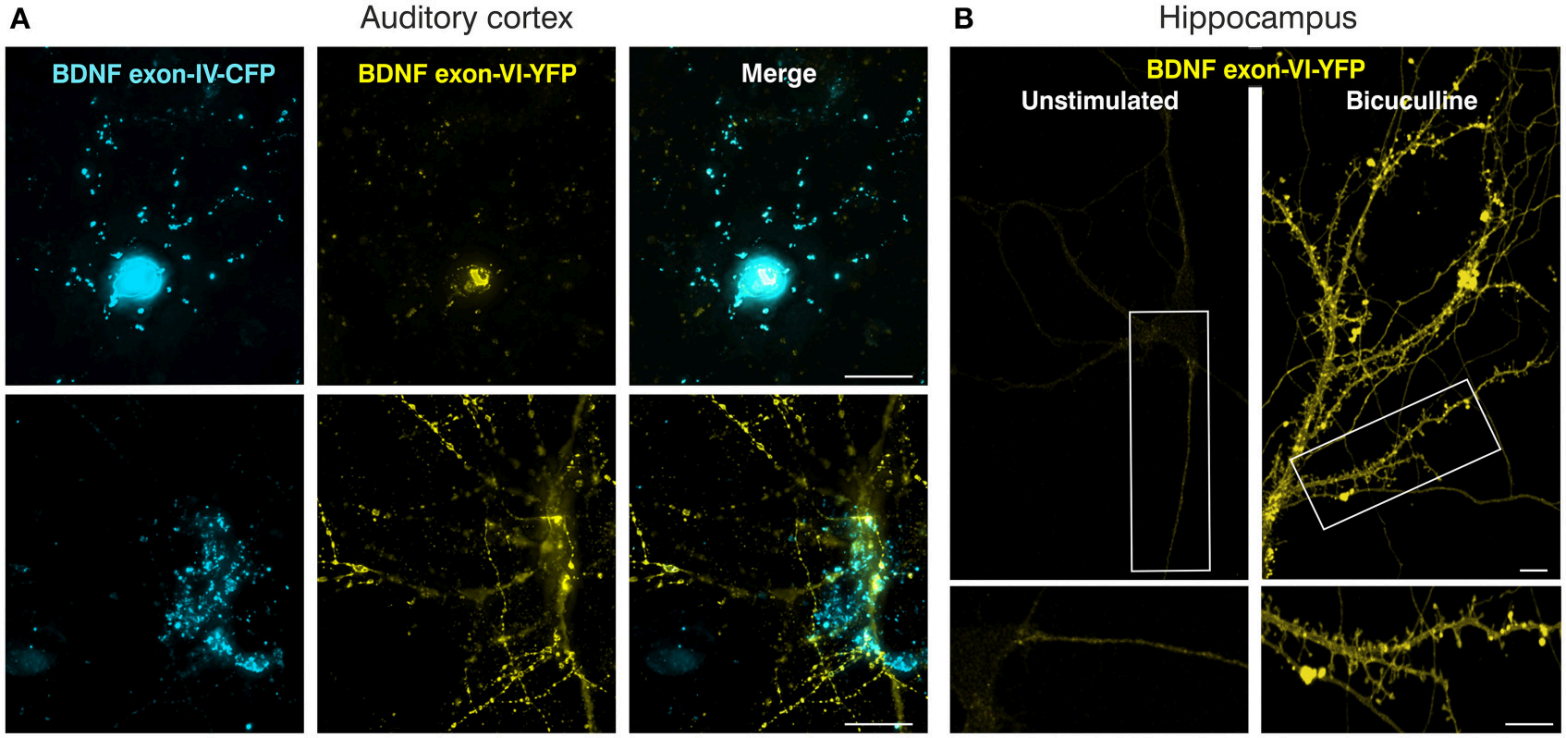
Verification of the BLEV double construct in primary neuronal cultures. **(A)** Mouse primary cortical cell culture (postnatal day 2) transfected with the BDNF double construct. Left panels: *Bdnf* exon-IV-CFP expression; middle panels: *Bdnf* exon-VI-YFP expression; right panels: merged images. Top and bottom panels represent two different neurons. Scale bar: 20 μm. **(B)** Transfected rat primary hippocampal neurons (embryonic day E18) without (left) or with bicuculline (right) stimulation showing *Bdnf* exon-VI-YFP expression. *Bdnf* exon-IV-CFP expression showed an increase preferentially in the cell body (Supplementary Figure 2A, arrows), while exon-VI-YFP was predominantly detected in proximal and distal regions of the neurites (Supplementary Figure 2B, lower panels). Scale bars: 10 μm.

The feasibility of this approach was first tested by transfecting the neuroglioblastoma cell line SK-N-BE, with either the exon-IV-CFP (Figures 1A upper panel, **C**) or the exon-VI-YFP single construct (Figures 1A lower panel, **D**). Distinct SK-N-BE cells exhibited either CFP or YFP expression (Figures 1C,D). To confirm whether BDNF protein is synthetized from these constructs, we performed ELISA assays to compare BDNF protein levels between untransfected and transfected cells (Figure 1E). Both single constructs clearly led to elevated levels of BDNF protein expression (blue column: *Bdnf* exon-IV-CFP (HA)-IX; yellow column: *Bdnf* exon-VI-YFP (cMyc)-IX) in comparison to untransfected cells treated with Lipofectamine (LTX) only (Figure 1E, gray column). The differences in BDNF protein expression between the two single constructs might be due to the different activation potential of the promoter regions of *Bdnf* exon-IV and -VI (Baj and Tongiorgi, 2009). Furthermore, a different transfection rate of the exon-IV-CFP and the exon-VI-YFP single constructs cannot be excluded as the cells are not stably transfected. Next, we tested the capacity of the double construct to visualize differential expression of *Bdnf* exon-IV-CFP and *Bdnf* exon-VI-YFP in distinct cells (Figure 1F). Transfection of the SK-N-BE cells lead either to the expression of CFP (Figure 1F, left panel, open arrows), YFP (Figure 1F, middle panel, open arrows), or both fluorescence proteins (Figure 1F, closed arrows) confirming that parallel observation of the two non-coding exons expressed from the double construct is feasible. When the double construct (Figure 1B) was transfected into primary neuronal cultures of the auditory cortex (AC) (Figure 2A), exon-IV-CFP preferentially localized to somata, with comparably limited targeting to dendrites (Figure 2A, upper and left panels). Exon-VI-YFP, in contrast, was predominantly found in dendrites, and only rarely in the soma (Figure 2A, compare upper and lower panel). This differential distribution of exon-IV-CFP and exon-VI-YFP suggests that the GAP43 membrane-tag does not interfere with the visualization of the fluorescence proteins as observed previously (Liu et al., 1994). Next, transfected primary cultures of hippocampal neurons were treated with the GABA_A_ receptor antagonist bicuculline (50 μM), 4-aminopyridine (2.5 mM), and glycine (10 μM) (Figure 2B), to elevate spontaneous activity which upregulates BDNF expression (Kim et al., 2012). This led to a clear increase in localization of exon-VI-YFP in dendrites and dendritic spines (Figure 2B). Distinct results were obtained for exon-VI-YFP and exon-IV-CFP, with the latter construct showing following bicuculline treatment an increased expression preferentially in the cell body (Supplementary Figure 2A, arrows), while exon-VI-YFP was predominantly detected in proximal and distal regions of the neurites (Supplementary Figure 2B, lower panels). These results confirm that in primary cultures of hippocampal neurons BDNF derived from both exons is differentially targeted to distinct subcellular compartments (Vaghi et al., 2014).

In summary, we introduce a new gene construct, BLEV, allowing observation of changes in exon-IV and exon-VI *Bdnf* promoter usage in response to defined stimuli *in vitro*.

### Generation of a reporter mouse model to monitor exon-IV-CFP and exon-VI-YFP

**Figure 3:**
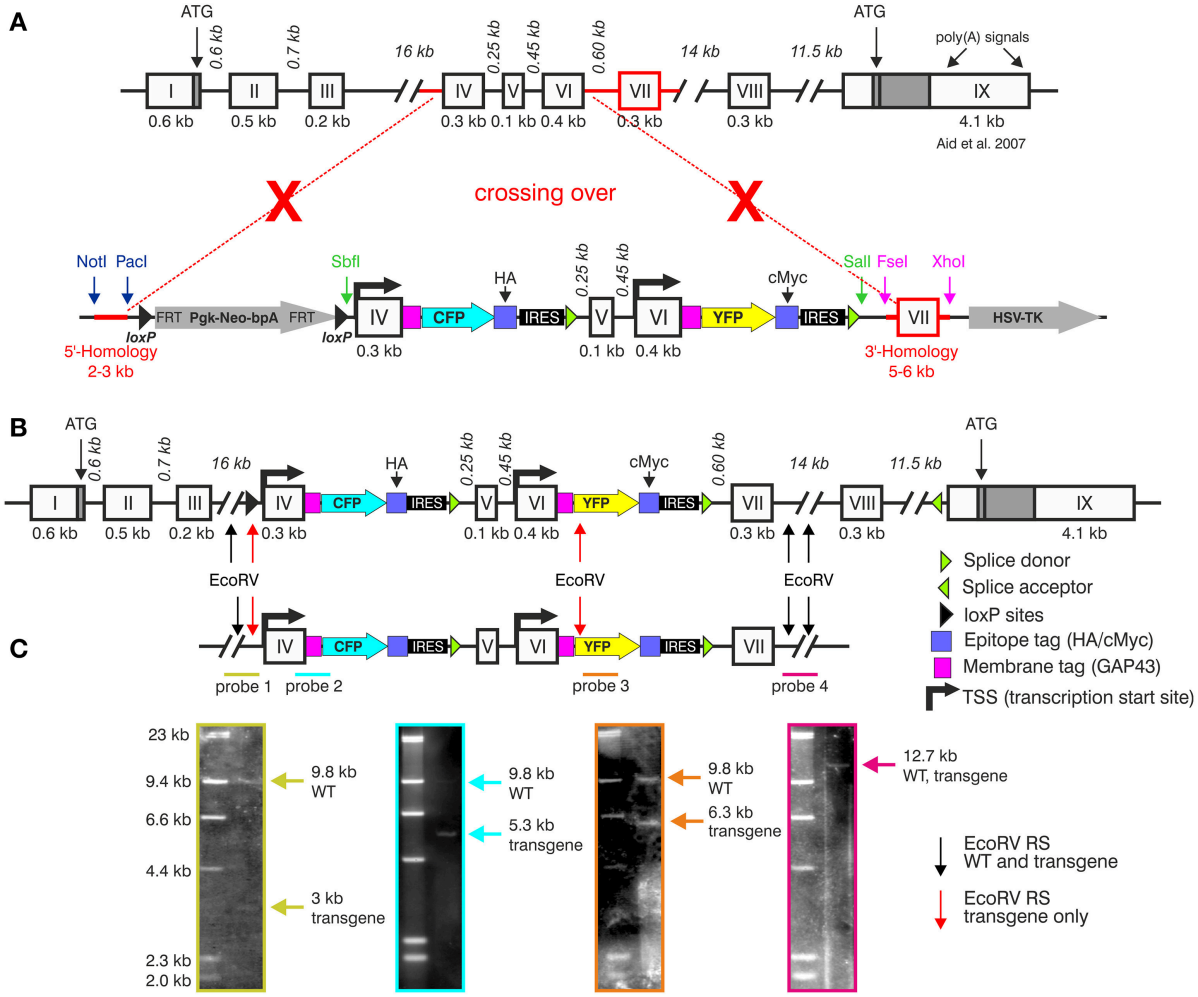
Generation of the BLEV reporter mouse. **(A)** Schematic drawing of the *Bdnf* gene (Aid et al., 2007) and the BDNF vector construct used for homologous recombination. Crossing-over events are indicated by red dotted lines and crosses. The 5' and 3' parts for homologous recombination are marked in red. **(B)** *Bdnf* gene construct depicting insertion sites of the BLEV construct. Arrows indicate the EcoRV restriction sites (RS) used for Southern blot. Black arrows indicate EcoRV RS cutting the WT and the transgenic sequence, red arrows cut only the transgenic sequence. **(C)** Southern blot of a positively transfected ES cell clone. The positions of the four Southern blot probes (colored lines: green, blue, orange, and pink) are shown under the schematic drawing of the BDNF construct. Probe 1, specific for the 5′-end: WT band 9.8 kb, transgenic allele 3 kb; probe 2, covering parts of exon-IV transgene: WT band 9.8 kb, transgenic allele 5.3 kb; probe 3, covering parts of the exon-VI transgene: WT band 9.8 kb, transgenic allele 6.3 kb; probe 4, specific for the 3′-end: WT and transgenic allele 12.7 kb. Note that the size of the drawn construct and probes does not reflect the real size of the DNA fragments obtained by Southern blot. For original Blots see Supplementary Figure 1.

The BLEV construct was inserted into the genomic locus of *Bdnf* via homologous recombination in mouse embryonic stem cells to replace the region harboring exon-IV, -V, -VI, and -VII (between bp 21,000 and 30,485 GenBank ID AY057907) (Figures 3A,B). Fidelity of the targeting event was validated by Southern blots using internal as well as 5' or 3' external probes (Figure 3C). The genomic DNA was cut by EcoRV [Figure 3C, arrows indicate EcoRV restriction sites (RS)] for Southern blotting. The results confirmed the correct insertion within the genomic BDNF sequence using specific probes for the 5'-end (Probe 1, wildtype (WT) band 9.8 kb, transgenic allele 3 kb), the exon-IV transgene (Probe 2, WT band 9.8 kb, transgenic allele 5.3 kb), the exon-VI transgene (Probe 3, WT band 9.8 kb, transgenic allele 6.3 kb) and the 3'-end (Probe 4: WT and transgenic allele 12.7 kb), respectively (Figure 3C).

**Figure 4:**
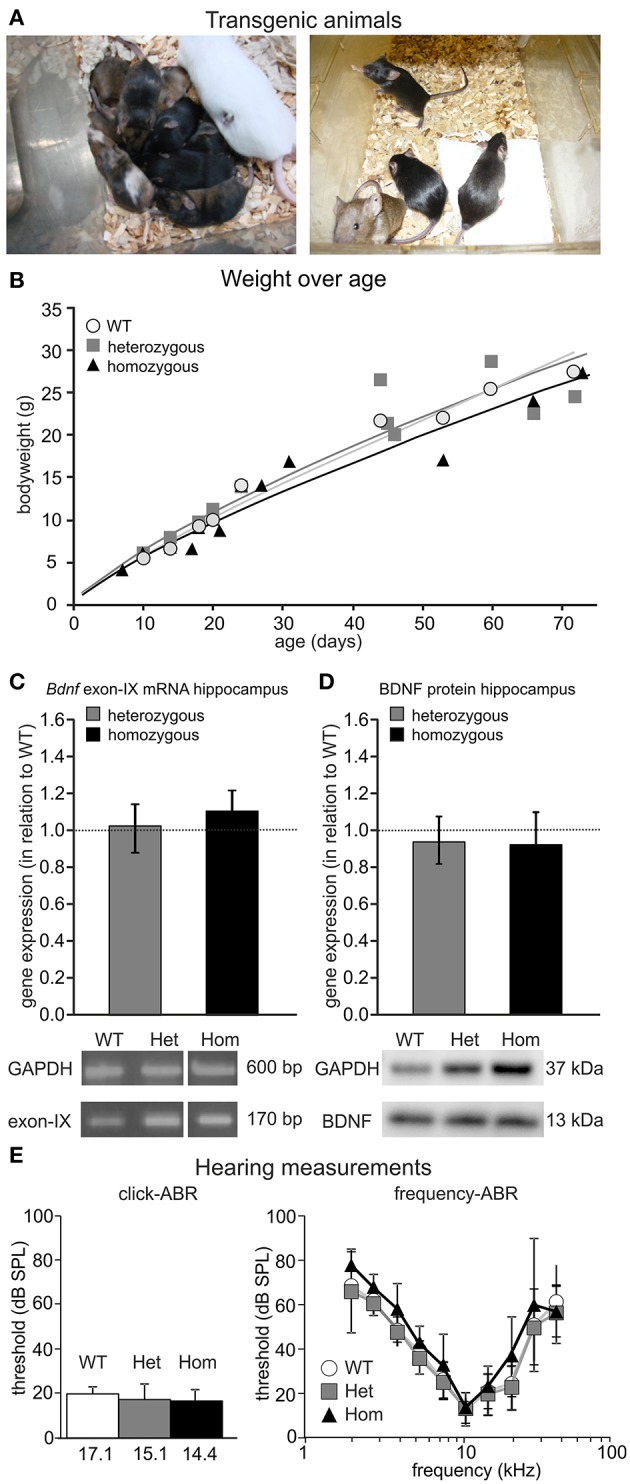
Phenotype of the BLEV mouse line. **(A)** Heterozygous BLEV mouse (white; left panel) and adult homozygous animals (right panel). **(B)** Comparison of the weight of the different genotypes (WT; het; hom). Individual symbols represent individual animals (WT *n* = 9, Het *n* = 11, Hom *n* = 11 animals). **(C)** Analysis of *Bdnf* exon-IX mRNA expression by RT-PCR in the hippocampus of BLEV mice for WT, heterozygous and homozygous animals. BDNF expression was normalized to the WT expression level (dotted line). No changes in the expression of untranslated *Bdnf* exons (I, II, III, IV, VI, IXA) were observed using RT-PCR (Supplementary Figure 3). Housekeeping gene: GAPDH. Data represented as mean ± SEM [1-way ANOVA: *F*_(2, 9)_ = 0.15 *p* = 0.86; *n* = 3 animals/genotype]. For original picture see Supplementary Figure 6A. **(D)** Analysis of BDNF protein expression by Western Blot in the hippocampus of BLEV mice for WT, heterozygous and homozygous animals. BDNF expression was normalized to the WT expression level. Housekeeping gene: GAPDH. Data represented as mean ± SEM [1-way ANOVA: *F*_(2, 12)_ = 0.20 *p* = 0.82; *n* = 4 animals/genotype]. For original blot see Supplementary Figure 6B. **(E)** Basic hearing function of BLEV mice for WT, heterozygous and homozygous animals. Thresholds for auditory brainstem responses (ABR) to click-stimuli (left panel) and varying pure tone frequencies (right panel). Data represented as mean ± SD [click-ABR: 1-way ANOVA: *F*_(2, 26)_ = 1.988 *p* = 0.16; f-ABR: 2-way ANOVA: *F*_(18, 2665)_ = 0.50 *p* = 0.96; WT *n* = 10; het *n* = 15; hom *n* = 5 animals].

Chimeras were carried by foster mothers and bred over two generations to obtain homozygous BLEV reporter mice. All three genotypes (WT, heterozygous and homozygous animals) were obtained at the expected Mendelian ratio (Figure 4A), were fertile, and had a normal life span. Also, no differences in body weight, known to occur upon BDNF deficits (for reviews see: (Rios, 2013, 2014) were observed between WT, heterozygous, and homozygous mice (Figure 4B). Furthermore, using RT-PCR (Figure 4C) and Western blot (Figure 4D), no differences in the hippocampal levels of mRNA containing *Bdnf* exon-IX, the only protein-encoding region of BDNF, or BDNF protein between adult WT, heterozygous and homozygous BLEV mice were detected (Figures 4C,D). No changes in the expression of untranslated *Bdnf* exons (I, II, III, IV, VI, IXA) were observed using RT-PCR (Supplementary Figure 3). The untranslated exons V, VII, and VIII were below the detection level of RT-PCR in the hippocampus.

Previous findings demonstrated mild but significant hearing loss in mice with BDNF deletion in the cochlea (Zuccotti et al., 2012). We compared threshold of ABRs of heterozygous and homozygous BLEV mice to WT controls for click-stimuli (Figure 4E, click-ABR) and frequency-dependent ABRs (Figure 4E, frequency-ABR). No apparent differences between the genotypes were observed. Additionally, BLEV mice did also not show any circling behavior indicating impaired BDNF expression in the vestibular system (Kaiser et al., 2001; Zuccotti et al., 2012).

Taken together, normal BDNF levels in the CNS and the lack of BDNF-specific phenotypes suggest that BLEV reporter mice retain physiological BDNF expression and functionality.

### Exon-IV-CFP and exon-VI-YFP expression in BLEV reporter mice co-localize with endogenous BDNF in neuronal, glial, and vascular cells

**Figure 5:**
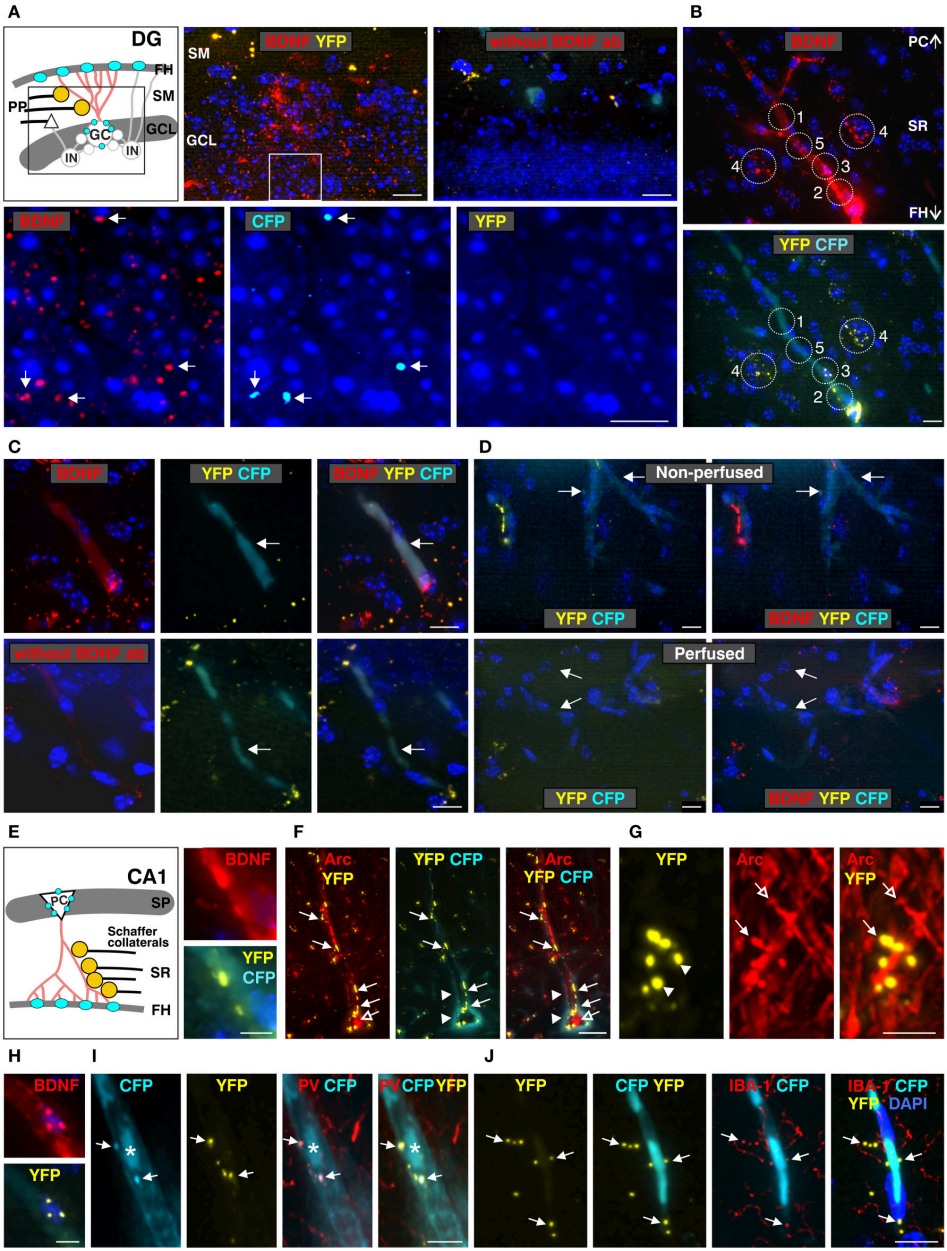
Co-localization of anti-BDNF antibody with *Bdnf* exon-IV-CFP and *Bdnf* exon-VI-YFP in the dentate gyrus (DG, **A**) and the CA1 region **(B–J)** (see Supplementary Video 1). **(A)** Top panel: schematic view of the granule cell layer (GCL) and the stratum moleculare (SM) of the DG. Low-power examination depicts BDNF-IR within the GCL of the DG (boxed area in sketch). No labeling was seen when anti-BDNF was omitted. Bottom panel: high-power examination from boxed area shows BDNF to co-localize with a few CFP-positive dots (arrows) but not with YFP-positive dots. Scale bars: 100 μm. **(B)** BDNF-IR in 5 different characteristic regions at the level of the stratum radiatum (SR) co-localized with *Bdnf* exon-VI-YFP and *Bdnf* exon-IV-CFP, see also Supplementary Figure 4. Scale bars: 100 μm. **(C)** BDNF-IR in blood vessel (arrows) co-localized with *Bdnf* exon-IV-CFP but not with *Bdnf* exon-VI-YFP. No BDNF-IR is observed when BDNF antibody is omitted (lower panel). Corresponds to area 1 and 5 in **(B)**. Scale bars: 20 μm. **(D)** *Bdnf* exon-IV-CFP labeling in blood vessels (arrows) of non-perfused BLEV mice (upper panel) was lost when animals were perfused (lower panel). Scale bars: 20 μm. **(E)** Schematic overview of the SR in the CA1 region. BDNF-IR co-localized with *Bdnf* exon-VI-YFP close to a *Bdnf* exon-IV-CFP positive capillary in the fissura hippocampalis (FH). Corresponds to 3 and 4 in **(B)**. Scale bar: 10 μm. **(F)** YFP-positive contacts (closed arrows, see also Supplementary Figure 5) on glutamatergic, Arc-positive dendrites (open arrows) of CA1 neurons embedded within the highly vascularized FH (arrowheads). Scale bar: 20 μm. **(G)** High-power examination of potential Schaffer collateral (SC) terminals labeled with *Bdnf* exon-VI-YFP and contacting postsynaptic Arc-positive spines in the SR (compare open and closed arrows). Scale bar: 5 μm. **(H)** BDNF-IR co-localized with *Bdnf* exon-VI-YFP-positive puncta on *Bdnf* exon-IV-CFP-positive capillaries (CFP not shown). Corresponds to 2 in **(B)**. Scale bar: 10 μm. **(I)** *Bdnf* exon-VI-YFP-positive puncta close to an endothelial nucleus (asterisk) overlapping with parvalbumin (PV)-positive interneuron dendrites (arrows). Corresponds to 2 in **(B)**. Scale bar: 10 μm. **(J)** *Bdnf* exon-VI-YFP-positive puncta close to an endothelial nucleus overlapping with the microglia marker IBA-1 (arrows). Corresponds to 3 and 4 in **(B)**. Scale bar: 20 μm. GC, granular cell; IN, inhibitory interneuron; PC, pyramidal cell; PP, perforant path; SP, stratum pyramidale.

**Figure 6:**
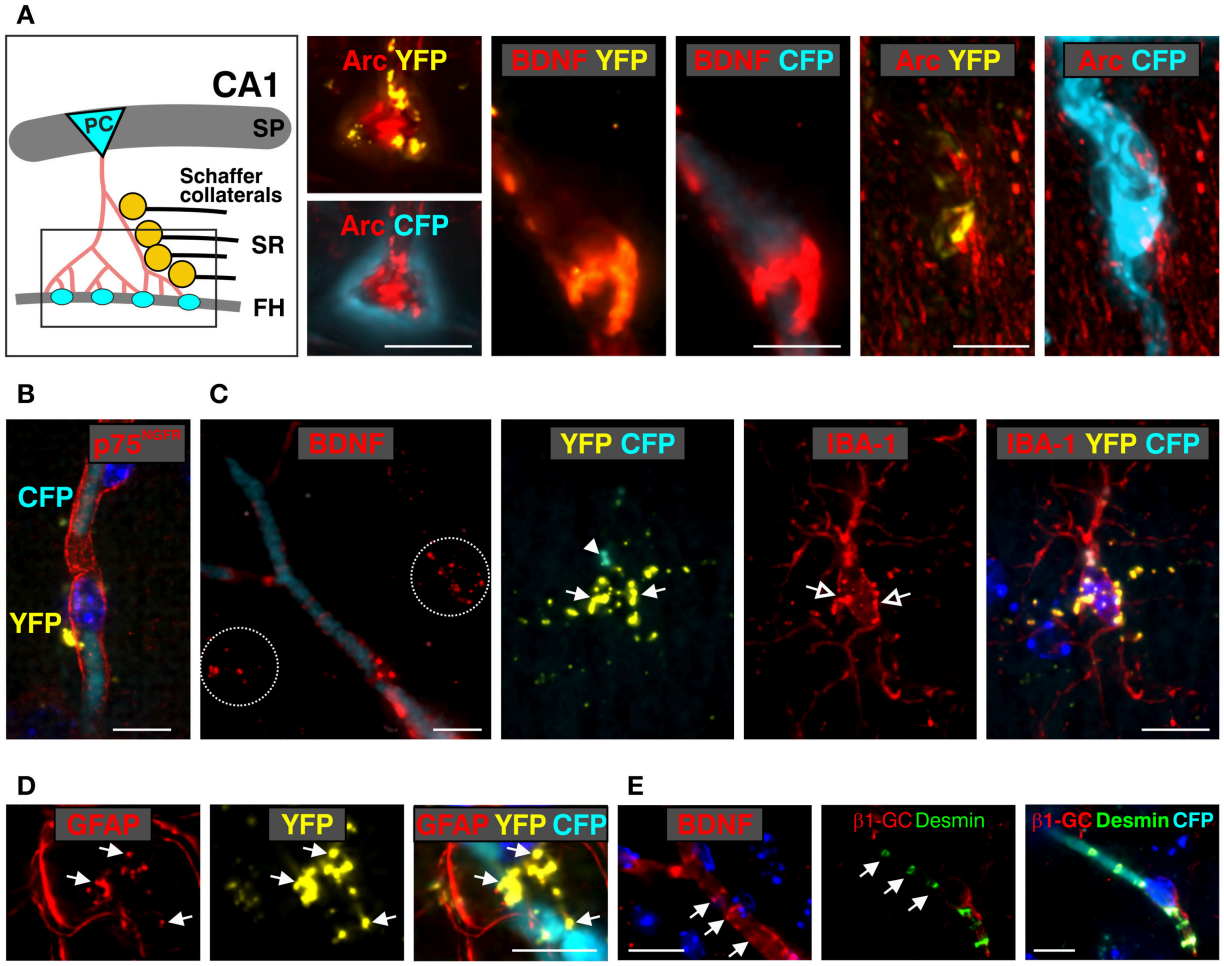
Co-localization of anti-BDNF with *Bdnf* exon-IV-CFP and *Bdnf* exon-VI-YFP in the stratum radiatum (SR) and the vasculature of the fissura hippocampalis (FH) (see Supplementary Video 2). **(A)** Schematic overview of the stratum radiatum (SR) region of the hippocampal formation. Arc-positive (red) pyramidal cell (PC) dendrites contact the vascularized fissura hippocampalis (FH) embedded within a *Bdnf* exon-IV-CFP-positive capillary bed (left). BDNF immunoreactivity (red) co-localizes with *Bdnf* exon-VI-YFP puncta within this region covering a *Bdnf* exon-IV-CFP-positive blood vessel (middle). In another view of the FH region, the *Bdnf* exon-VI-YFP puncta contacting Arc-positive dendrites deeply entering the basal membrane of a *Bdnf* exon-IV-CFP-positive blood vessel is shown (right). Scale bars: 10 μm. SP, stratum pyramidale. **(B)** *Bdnf* exon-VI-YFP contacting p75^NGF^ receptor-positive (red) endothelia cells of a *Bdnf* exon-IV-CFP-positive blood vessel. Nuclei are stained in dark blue with DAPI. Scale bars: 10 μm. **(C)** The characteristic BDNF immunoreactivity pattern (red, dotted circles) seen in the SR region close to blood vessels outlined with *Bdnf* exon-IV-CFP. *Bdnf* exon-VI-YFP-positive labeling (closed arrows) is observed close to an IBA-1-positive (red) microglia soma (open arrows) that itself targets *Bdnf* exon-IV-CFP at the microglia pole (closed arrowhead). Scale bars: 10 μm. **(D)** *Bdnf* exon-VI-YFP-positive pattern (closed arrow) observed in overlap with glial fibrillary acidic protein (GFAP)-labeled astrocyte endfeets close to a *Bdnf* exon-IV-CFP-positive blood vessel. Scale bars: 10 μm. **(E)** BDNF-IR (red, arrows) in endothelial cells in a pattern resembling localization of desmin (green, arrows), a marker of pericytes. Pericyte processes embrace the abluminal endothelium wall of an exon-IV-CFP-positive blood vessel encircled by β1-guanylyl cyclase (GC)-positive (red) smooth muscle cells. Scale bars: 10 μm.

To further validate the BLEV reporter mouse line we next compared exon-IV-CFP and exon-VI-YFP fluorescence signals with BDNF protein expression *in vivo*. These experiments were performed in hippocampal brain slices of homozygous BLEV reporter mice (Figures 5, 6). Hippocampal sections were stained with an antibody specific for the BDNF pro-domain (Dieni et al., 2012; Figures 5, 6, BDNF, see Supplementary Table 1). BDNF staining was compared to exon-IV-CFP and exon-VI-YFP signals in sections co-stained with markers for excitatory and inhibitory neurons, glial cells or vascular cells and correlated to previously described sites of BDNF expression (Figures 5, 6, see Supplementary Table 1) (for review see: Edelmann et al., 2014). In particular those described in distinct hippocampal cells (Danzer and McNamara, 2004; Danzer et al., 2008).

Examination of BDNF signals in the dentate gyrus (DG) at low-magnification revealed immunoreactivity (IR) in the supra- and infra-pyramidal blades of the granule cell layer (GCL, Figure 5A), as previously observed (Dieni et al., 2012). No labeling was detected when the BDNF antibody was omitted (Figure 5A, right upper panel). Under high magnification, we detected co-localization of BDNF with a few exon-IV-CFP positive dots in the GCL, but not with exon-VI-YFP (Figure 5A, lower panel, arrows). Moreover, BDNF-IR was observed in at least five different characteristic regions, as here shown for the SR at the level of the CA1, that were either co-labeled with exon-IV-CFP (Figure 5B, No. 1, Supplementary Figure 4), exon-VI-YFP (Figure 5B, No. 2–4), or neither of the fluorochromes (Figure 5B, No. 5; Supplementary Video 1, Supplementary Figure 4). Additionally, faint BDNF-IR was observed in blood vessels (Figure 5C) where it co-localized with exon-IV-CFP, but not exon-VI-YFP (Figure 5C, upper panel). No BDNF labeling was detected upon omission of the primary antibody (Figure 5C, lower panel). Related to previous observations of BDNF in circulating platelets (Chacón-Fernández et al., 2016), we also detected CFP fluorescence in vessels of non-perfused animals (Figure 5D, upper panel; Supplementary Video 1) in contrast to perfused animals, where platelets are expected to be washed out (Figure 5D, lower panel).

Common wiring of nerve and blood vessels has been suggested (Carmeliet and Tessier-Lavigne, 2005) but its monitoring has so far been elusive. Our analysis revealed a dot-like BDNF-IR, co-localized with exon-VI-YFP close to an exon-IV-CFP positive capillary embedded in the fissura hippocampalis (FH) (Figure 5E; Supplementary Video 2). YFP dots represent glutamatergic terminals contacting Arc-positive dendrites of CA1 neurons (Figures 5F, 6A; Supplementary Video 1). These dendrites of CA1 neurons are often embedded within the highly vascularized FH (Soriano and Frotscher, 1993). At higher magnification, it became evident that exon-VI-YFP labeled presumptive Schaffer collateral (SC) terminals contacted postsynaptic spines of Arc-positive dendrites in the stratum radiatum (SR) (Figure 5G; Supplementary Video 1). As only a few Arc-positive spines were contacted by exon-VI-YFP positive dots (Figure 5G, compare closed and open arrow), we surmise that these may correspond to activated synapses. Presynaptic expression of YFP could be further confirmed by co-localization of YFP with the presynaptic marker proteins VGLUT1, VGLUT2 and VGLUT3 (Supplementary Figure 5; Somogyi et al., 2004; Herzog et al., 2006). VGLUT1 and VGLUT3, described in mossy fiber terminals (Somogyi et al., 2004; Herzog et al., 2006), were found to be co-localized with YFP in the CA3 region (Supplementary Figures 5A,B). VGLUT2, described in perforant path terminals contacting dentate gyrus granular cells (Herzog et al., 2006), was found to be co-localized with YFP in the stratum moleculare (Supplementary Figure 5C). Interestingly, YFP also co-localized with VGLUT3 in the glomerular layer of the olfactory bulb, suggesting exon-VI-YFP to co-localize in glomerular layer-projecting cholinergic terminals (Case et al., 2017).

BDNF-positive dots co-localizing with exon-VI-YFP were also observed close to a nucleus of an endothelial cell (Figure 5H, magnified from Figure 5B, No. 3), as shown by co-staining with the p75^NGF^ receptor, an endothelial marker (Xu et al., 2008; Figure 6B). The characteristic exon-VI-YFP pattern close to endothelial nuclei was repeatedly found to either overlap with parvalbumin (PV), a marker of inhibitory neurons (Figure 5I; Supplementary Video 1), or IBA-1 (ionized calcium-binding adapter molecule 1), a marker of microglia (Frick et al., 2013; Figure 5J; Supplementary Video 1).

The BDNF-IR pattern typically seen close to blood vessels in the SR (Figures 5B, 6C) co-localized with exon-VI-YFP (Figure 6C, closed arrow) and exon-IV-CFP expressing presynaptic boutons (Figure 6C, arrowhead), both clearly overlapping with IBA1-labelled microglia (Figure 6C, open arrow; Supplementary Video 2). The exon-IV-CFP and exon-VI-YFP signals may represent SC terminals contacting microglia. In addition to microglia, the astrocyte specific marker GFAP (glial fibrillary acidic protein) also co-localized with exon-VI-YFP in close proximity to blood vessels (Figure 6D; Supplementary Video 2).

Finally, we observed BDNF-IR in capillary vessels, possibly within endothelial cells (Figure 5B, No. 5; Figure 6E), confirming previous observations (Donovan et al., 2000). Here, clusters of BDNF-IR were seen in zones (Figure 6E) where desmin positive pericyte processes variably surrounded the abluminal endothelial wall of an exon-IV-CFP positive blood vessel encircled by β 1-guanylyl cyclase (GC)-positive smooth muscle cells (Figure 6E, red; Supplementary Video 2).

These findings demonstrated several key points. First, wherever we detect exon-IV-CFP or exon-VI-YFP in the hippocampal path we also detect BDNF. Second, sites where BDNF is detected correspond to cell types previously shown to express BDNF. Third exon-IV-CFP and exon-VI-YFP containing splice variants are translated in non-overlapping locations on the cellular and subcellular level. Exon-IV-CFP is targeted to the somata of pyramidal, granule, or microglial cells, while exon-VI-YFP is detected in terminals of the tri-synaptic pathway as well as in the end-feet of microglia or astrocytes. Finally, BDNF in circulating blood is translated from exon-IV containing transcripts while BDNF in endothelial cells is probably translated neither from exon-IV nor from exon-VI containing transcripts.

In conclusion, the BLEV reporter mouse line allows observation of differences in exon-IV and -VI specific BDNF expression in distinct neuronal, glial, and vascular cells using high-resolution fluorescence microscopy.

### Activity-dependent up-regulation of exon-IV-CFP and exon-VI-YFP expression in BLEV reporter mice after injection with kainic acid

**Figure 7:**
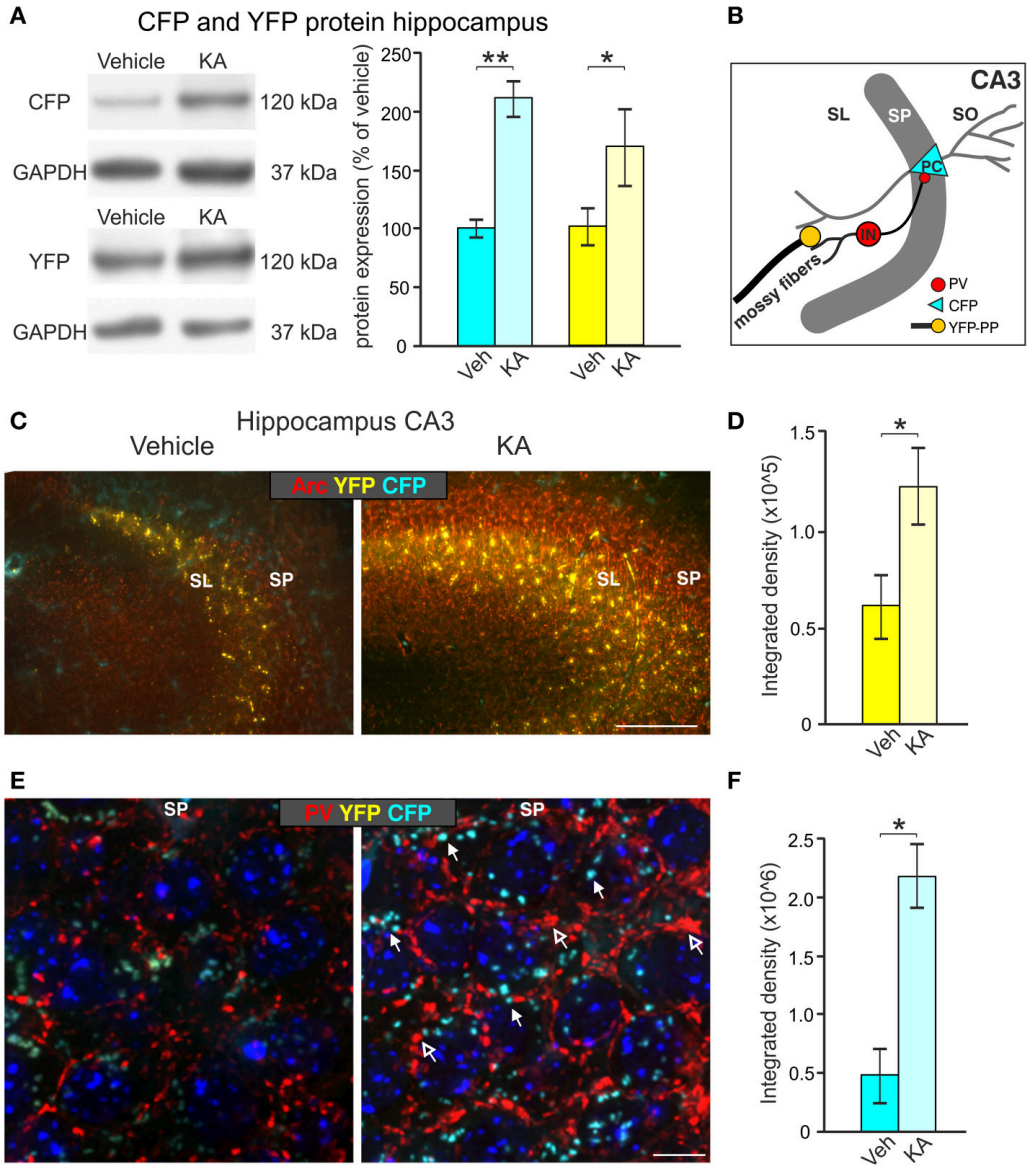
Induction of CFP and YFP expression by kainic acid treatment (KA). **(A)** Western blot analysis of hippocampal tissue lysates from animals injected with vehicle or KA. Left panel: representative Western blot against CFP (top) and YFP (bottom). Right panel: densitometric quantification shows significant up-regulation of CFP and YFP expression after KA treatment. Data represented as mean ± SEM normalized to vehicle treated controls, *n* = 3–4 animals/group (CFP: 1-sided Student's *t*-test: *t* = 6.25 DF = 4 *p* = 0.002; *n* = 3 animals/group; YFP: 1-sided Student's *t*-test: *t* = 1.99 DF = 6 *p* = 0.04; *n* = 4 animals/group). For original blot see Supplementary Figure 6C. **(B)** Schematic overview of the hippocampal CA3 region depicting the assumed locations of altered CFP, YFP and Parvalbumin (PV) expression. **(C)** CFP and YFP fluorescence in brain slices of vehicle- and KA-treated animals co-labeled with the cytoskeletal activity-regulated gene Arc. Clear upregulation of Arc (red) mainly in the SP and YFP mainly in the SL can be seen in the hippocampal CA3 region of KA-treated animals in comparison to vehicle-treated mice. Scale bars: 100 μm. **(D)** Quantification of the integrated density of YFP which is increased after KA injection (YFP: 1-sided Student's *t*-test: *t* = 4.14 DF = 3 *p* = 0.0128; *n* = 3 animals/group). **(E)** An increase in perisomatic CFP (closed arrows) and PV (red, open arrows) signals can be seen in the SP of the hippocampal CA3 region in KA-treated animals in contrast to vehicle-treated mice. Scale bars = 10 μm. **(F)** Quantification of the integrated density of CFP which is increased after KA injection (CFP: 1-sided Student's *t*-test: *t* = 2.32 DF = 4 *p* = 0.0405; *n* = 2–3 animals/group). SL, stratum lucidium; SP, stratum pyramidale; SO, stratum oriens; PC, pyramidal cell; PP, perforant path; IN, interneuron.

To investigate whether the BLEV reporter mouse line is suitable to study activity-dependent alterations of *Bdnf* exon-IV and *Bdnf* exon-VI usage *in vivo*, we validated CFP and YFP expression following the injection of kainic acid (KA) into homozygous BLEV reporter mice. KA has been shown to increase BDNF expression in the hippocampus by agonizing glutamate receptors (Zafra et al., 1990; Sathanoori et al., 2004; Chiaruttini et al., 2008) and to activate translation of exon-IV and -VI containing *Bdnf* mRNA (Metsis et al., 1993; Tao et al., 1998; Pattabiraman et al., 2005; Aid et al., 2007; Chiaruttini et al., 2008). Two hours following intra-peritoneal injection of KA (12 mg/kg), significant up-regulation of CFP and YFP protein was observed in the hippocampus in comparison to vehicle-treated animals using Western blot (Figure 7A).

We next focused on dentate mossy fiber boutons contacting hippocampal CA3 pyramidal neurons (Figure 7B), where activity-dependent expression of BDNF was previously described (Danzer and McNamara, 2004; Danzer et al., 2008; Dieni et al., 2012). Brain slices of vehicle- and KA-treated homozygous BLEV mice were co-labeled with antibodies against the cytoskeletal activity-regulated gene Arc, a protein essential for BDNF-dependent consolidation of LTP (Soulé et al., 2006; Messaoudi et al., 2007; Nair et al., 2017). Low power examination of deconvoluted high-resolution fluorescence stacks revealed a strong up-regulation of *Bdnf* exon-VI (YFP) fluorescence in KA-treated animals in mossy fiber projection fields of CA3 pyramidal neurons (Figure 7C), suggesting a KA-induced recruitment of *Bdnf* exon-VI derived BDNF in perforant path dendrites. We also co-stained sections with parvalbumin (PV) antibodies, a marker for fast inhibitory circuits suggested to be shaped by BDNF (Yamada and Nabeshima, 2003; Messaoudi et al., 2007; Minichiello, 2009; Waterhouse et al., 2012). Low power examination of deconvoluted high-resolution fluorescence stacks revealed an upregulation of *Bdnf* exon-IV (CFP) at the level of CA3 pyramidal neurons which correlated with elevated PV levels in dendritic pre-synaptic terminals surrounding pyramidal neurons in a perisomatic distribution (Klausberger et al., 2003; Somogyi et al., 2014; Figure 7E, CFP: closed arrow, PV: open arrows). Quantification of the integrated density of YFP and CFP in the CA3 region approved a significant increase after KA injection (Figures 7D,F). The quantitative change of YFP and CFP expression in the hippocampus shown by Western blot (Figure 7A, right panel), together with the parallel qualitative and quantitative change in YFP and CFP expression in the CA3 regions (Figures 7C–F), suggest that BLEV reporter mice are suitable to detect activity-dependent exon-specific changes under healthy and pathological conditions.

## Discussion

The present study proposes the BLEV system as a novel tool for monitoring BDNF expression based on the localization of mRNA containing the activity-dependent exons-IV and -VI of cellular and sub-cellular levels *in vitro* and *in vivo* through cyan fluorescent protein (CFP) and yellow fluorescent protein (YFP). We introduce the BLEV construct and mouse line as a tool to (i) analyze promoter activation or targeting characteristics of *Bdnf* exon-IV and -VI splice-variants *in vitro*; (ii) analyze cell specific differences in *Bdnf* exon-IV and -VI transcript usage in neuronal and non-neuronal cells; and (iii) detect activity-dependent and cell-specific BDNF splice-variant usage under healthy and pathological conditions *in vivo*.

### BLEV mice as a tool to investigate promoter activation patterns of BDNF

Analysis of the gene-structure of BDNF reveals the presence of nine exons where the eight upstream exons (I-VIII) are alternatively spliced to the ninth exon (IX), corresponding to the only exon encoding the BDNF protein (Timmusk et al., 1993; Aid et al., 2007; Figure 3A). The functions of the eight untranslated *Bdnf* exons (I-VIII) each containing a different promoter region is still unclear (Aid et al., 2007). Several studies investigated the different *Bdnf* exons and their activity-dependent activation (Timmusk et al., 1993; Lauterborn et al., 1996; Shieh et al., 1998; Takeuchi et al., 2000, 2002; Zha et al., 2001; Fang et al., 2003; Sathanoori et al., 2004; Sakata et al., 2009). Based on these studies, it was suggested that these promoters show a spatially distinct expression pattern and are regulated in a stimulus- and activity-dependent manner (Timmusk et al., 1993; Aid et al., 2007). To our knowledge, however, BDNF expression initiated from the untranslated *Bdnf* exons-IV and -VI containing transcripts has never been addressed in parallel *in vivo*. Therefore, cell-specific differences in exon-IV and -VI promoter activation in the mature system in response to external stimuli have so far never been detected under physiological conditions. The observed transcript specific expression corresponding to either of the distinct splice-variants or both, detected in a neuroglioblastoma cell line suggests that the BLEV construct may be useful to identify subtle and complex promoter activation patterns (Figure 1). While it is well established that activation from exon-IV promoter occurs following elevated levels of calcium (Ca^2+^) (West et al., 2014), the details under which conditions this occurs, e.g., through glutamate-induced NMDA receptors, voltage gated Ca^2+^ channels (VGCCs) or intracellular Ca^2+^ stores is still elusive. It is also not understood if neuronal, glial or vascular cells exhibit differences in second messenger cascades acting on specific promoter sites (e.g., CaMKII or CaMKIV, MAP kinase) (West et al., 2001; Takeuchi et al., 2002; Tao et al., 2002; Chen et al., 2003). While e.g., *Bdnf* exon-IV in platelets is suggested to be activated through store-operated Ca^2+^ channels (Chacón-Fernández et al., 2016), the mechanism associated with promoter activation remains elusive. An even greater complexity is observed regarding the *Bdnf* exon-VI promoter that is activated only modestly by neuronal activity (Timmusk et al., 1993; Aid et al., 2007), but is indirectly activated by neuronal activity through binding to the AP-1 family transcription factor site (Tuvikene et al., 2016). Here, BDNF-autoregulatory loops, acting via its promoters (Bambah-Mukku et al., 2014; Harward et al., 2016) may also be involved. The BLEV reporter system now allows investigation of this hypothesis *in vitro* or *in vivo*.

Kainic acid (KA), shown to activate glutamate receptors in hippocampal neurons (Zafra et al., 1992; Sathanoori et al., 2004; Chiaruttini et al., 2008), not only elevates exon-IV- and exon-VI- derived BDNF expression in hippocampal tissue but does so in a cell-specific manner (Figure 7), indicating that the activity-driven activation of BDNF promoters-IV and -VI is not impaired by the BLEV construct. This experiment also anticipates the usage of the BLEV construct to investigate not only cell specific differences in promoter usage but also the predicted complex interplay between transmitter-induced changes in BDNF expression and its reciprocal effects on its receptor TrkB (Flavell and Greenberg, 2008; Sakata et al., 2009; Lu et al., 2010). Here the perspective that the BLEV construct can be viewed using two-photon microscopy (Thaler and Vogel, 2006) following manipulations of promoter activation under different conditions is most promising.

In this context, the demonstration of differences in intraneuronal targeting of BDNF transcripts between *in vitro* and *in vivo* conditions observed in the present study is particularly interesting. Accordingly, *Bdnf* exon-IV and -VI were found in primary neuronal cultures to be targeted mainly to the soma and dendrites, respectively, as described previously (Chiaruttini et al., 2008; Baj et al., 2011; Vaghi et al., 2014). In the mature BLEV mouse model a differential intraneuronal targeting of BDNF transcripts derived from exon-IV-CFP to the soma and exon-VI-YFP to terminals (Chiaruttini et al., 2008; Baj et al., 2011; Vaghi et al., 2014) could not only be demonstrated for projecting neurons but interestingly also for microglia (Figures 5J, 6C). Importantly, however, and different from previous suggestions, we did not observe exon-VI-YFP signals in dendrites of e.g., the CA1 region (Figures 5F,G). Whether these observations support the previously suggested elusive anterograde transport of BDNF transcripts and its restricted presynaptic BDNF release (Dieni et al., 2012) needs further investigation. Indeed, we cannot entirely exclude that *Bdnf* transcripts may be differentially compartmentalized depending on the type of stimulus and time course, as shown previously (Chiaruttini et al., 2008).

This observation is of crucial interest since trafficking of exon-VI derived BDNF to nerve terminals is thought to be disturbed in animals or humans carrying the BDNF^Val66Met^ allele, causing a mutation linked to cognitive deficits (Baj et al., 2013; Mallei et al., 2015).

### BLEV mice as a tool to investigate cell-specific and transcript-specific differences of BDNF expression

The low abundance of BDNF in the mature CNS currently hampers investigation of cell-specific BDNF expression differences in the healthy or diseased mature brain (Dieni et al., 2012). We have shown that the localization of BDNF via specific antibodies overlaps with either exon-IV-CFP or exon-VI-YFP signals in neuronal, glial, or vascular compartments within the hippocampus (Figure 5). Importantly, the identified BDNF patterns correspond to previously observed regions of BDNF expression in neuronal or non-neuronal cells (Dieni et al., 2012; Chacón-Fernández et al., 2016). Thus our data imply that for example BDNF translation in the soma of hippocampal pyramidal cells (Danzer et al., 2008) results from *Bdnf* exon-IV splice variants, while BDNF translation in tri-synaptic hippocampal terminals (Danzer et al., 2008; Dieni et al., 2012) is generated by *Bdnf* exon-VI splice variants (Figures 5B, 6; Supplementary Video 1). In addition, expression of BDNF in astrocytes or microglia (Snapyan et al., 2009; Parkhurst et al., 2013), predicted to participate in the recruitment of blood vessels during complex homeostatic changes in plasticity (Edelmann et al., 2014, 2017), may be driven by exon-IV in the glial soma or by exon-VI when released from glial end-feet structures (Figures 6C,D; Supplementary Video 1). The BLEV reporter mice may thus provide a new resource to investigate to what extent the BDNF^Val66Met^ polymorphism, linked to cognitive deficits, may not only affect trafficking in neuronal (Baj et al., 2013; Mallei et al., 2015) but possibly also in microglial cells. Moreover, our results in BLEV mice indicate for the first time that BDNF is also present in mouse platelets, as previously described in humans and rats (Chacón-Fernández et al., 2016). How exon-IV derived BDNF in platelets can communicate through the blood-brain barrier that is maintained by endothelial tight junctions, pericytes, or astrocytic end-feet (Marosi and Mattson, 2014) could be part of future studies in BLEV mice. Finally, BDNF in endothelial cells, identified through co-localization of BDNF with p75^NGFR^ (for review see: Donovan et al., 2000; Marosi and Mattson, 2014), appears to be neither generated by exon-IV nor by exon-VI promoters (Figures 5B, 6B).

Conclusively, BLEV mice may thus be the first animal model enabling the parallel monitoring of BDNF expression differences in neurons, glial cells, and capillaries, including subcellular targeting in neurons and glial cells.

### BLEV mice as a tool to investigate stimulus-driven cell-specific and transcript-specific differences of BDNF in healthy and pathological conditions *in vivo*

The analysis of the role that BDNF plays for the nervous system has until now mainly focused on *in vitro* studies or BDNF knock-out mouse models. BDNF knock-out mice die postnatally (Ernfors et al., 1995) and therefore escape investigations of BDNF's roles in the mature system, particularly of those in response to changes in neuronal activity. However, the presently existing mouse models have indicated important phenotypic differences in the absence of BDNF signaling in the mature system. Accordingly, heterozygous BDNF knock-out animals show anxiety-like behavior (Chourbaji et al., 2008) or signs of obesity (Rios et al., 2001; Vanevski and Xu, 2013). Mice with a point mutation in the BDNF receptor TrkB gene exhibit progressive hearing loss (Postigo et al., 2002), while mice with a conditional deletion of BDNF from cortical neurons show a severe reduction of dendritic contacts (Rauskolb et al., 2010). A first hint for a crucial role of distinct BDNF transcripts in control of cortical inhibition, aggressive behavior (Lyons and West, 2011; Hill et al., 2016; Maynard et al., 2016), or e.g., in the induction of depression was obtained in BDNF mouse models in which one of its promoters had been impaired (Hong et al., 2008; Sakata et al., 2009). A further indication that intracellular targeting of BDNF transcript defines e.g., cognitive competence has been observed in BDNF^Val66Met^ mutants (Mallei et al., 2015). So far, however, none of these studies can explain why different activity-dependent BDNF promoters may provide advantages over usage of a single promoter.

Various studies have confirmed the requirement of glucocorticoid signaling in selected networks to support morphological changes at synapses (Cheng et al., 2012; Liston et al., 2013; Arango-Lievano et al., 2015) and an activity-dependent process to provide information about the appropriate context (De Kloet et al., 2014; Jeanneteau and Arango-Lievano, 2016). Indeed, previous hypotheses considered that activity-dependent BDNF expression provides context-dependent information for GR-mediated task-evoked plasticity changes to be associated with memory formation (Jeanneteau and Arango-Lievano, 2016). Up to now, however, it was not possible to directly observe activity-driven BDNF transcription. With the BLEV mouse, we now provide a suitable new tool for this purpose. In contrast to existing BDNF-GFP reporter mice (Guillemot et al., 2007), the BLEV model provides the advantage to allow visualization of different BDNF transcripts following activity and thereby to specifically monitor stimulus-driven activation patterns in networks. As a prerequisite to detect stimulus-driven BDNF transcript changes, we had to assure that gene replacement within the *Bdnf* locus did not interfere with the normal expression of the BDNF protein. BLEV mice were confirmed to express unchanged levels of BDNF and showed normal weight, lifespan, fertility, and function of the audio-vestibular sensory system. This is particularly important with regard to the crucial role BDNF plays for normal cognitive function (reviews: Minichiello, 2009; Lu et al., 2014; Leal et al., 2015), neurogenesis (Kheirbek et al., 2012; Waterhouse et al., 2012), energy homeostasis and pattern segregation (for review see: Bramham and Messaoudi, 2005; Rauskolb et al., 2010; Park and Poo, 2013; Marosi and Mattson, 2014; Turrigiano, 2014; Bothwell, 2016; Mitre et al., 2017). The lack of a changed phenotype thus qualifies BLEV reporter mice as new tool to investigate how expression of exon-IV and -VI derived BDNF changes after altered input activity in the different neuronal, glial or vascular cells during modulation of the aforementioned processes. This includes the use of BLEV mice for examining potential therapies in various disease models, where dysregulation of BDNF expression is predicted to contribute to the pathology, such as depression, epilepsy, or Alzheimer's, Huntington's, and Parkinson's disease (Bibel and Barde, 2000; Ginsberg et al., 2017).

As a first attempt to test if BLEV mice enable identification of activity-dependent adaptations in central networks under healthy or pathological conditions we shall focus on known plasticity paradigms in the hippocampus. The hippocampus is the region with highest levels of BDNF expression (Nawa et al., 1995; Conner et al., 1997), where it is predicted to play crucial roles for accentuating behaviorally important sound signals (Kilgard and Merzenich, 1998; Sadaghiani et al., 2009; Kraus and White-Schwoch, 2015; Weinberger, 2015). The precise organization of the peripheral and central auditory system together with functionally and molecularly established protocols (Rüttiger et al., 2017) to induce long-lasting plasticity changes related to memory (for review see: Knipper et al., 2013; Singer et al., 2013), provides an excellent model to investigate sound-induced activation patterns of *Bdnf* transcripts with the help of BLEV mice.

## Conclusion

In the present study, a transgenic system for ***B****DNF-****l****ive-****e****xon-****v****isualization* (BLEV) has been generated enabling the detection of activity-dependent BDNF translation from *Bdnf* exon-IV and -VI containing transcripts through CFP and YFP fluorescent proteins, respectively. The present study confirms (i) that transfection of different cell lines with the BLEV construct enables the tracking of intraneuronal targeting differences of the BDNF splice variants; (ii) that insertion of the BLEV construct into the genomic locus of *Bdnf* via homologous recombination resulted in healthy homozygous BLEV reporter mice without any apparent phenotypic changes and with normal levels of BDNF expression; (iii) that CFP and YFP fluorescence in BLEV mice overlaps with BDNF protein expression in neuronal, glial, and vascular locations; and (iv) that the BLEV construct allows identification of elevated *Bdnf* exon-IV-CFP and exon-VI-YFP expression levels following glutamate receptor activation *in vivo*. BLEV reporter mice can now be used to trace the potential role of activity-dependent BDNF promoter usage for providing context-specific information during task-specific memory formation.

## Author contributions

WS, RP-W, H-SG, TO, and MK conceptualization. WS, H-SG, MM, LR, and MK analysis. WS, H-SG, MM, GL, CBD, ILS, GB, EP, ET, KR, and TO investigation. WS, RP-W, MM, LM, PE, UZ, LR, TS, and MK writing. PR, TO, LR, and MK supervision. JH, LM, JS, PR, UZ, LR, and MK review and editing.

### Conflict of interest statement

The authors declare that the research was conducted in the absence of any commercial or financial relationships that could be construed as a potential conflict of interest.
