# Supplementary material for: BDNF-Live-Exon-Visualization (BLEV) Allows Differential Detection of BDNF Transcripts in vitro and in vivo
Source: Front Mol Neurosci. 2018 Sep 27;11:325. doi: 10.3389/fnmol.2018.00325 (PMC6170895; doi:10.3389/fnmol.2018.00325)
Supplement: Supplementary file 3 [file Data_Sheet_1.docx]

**
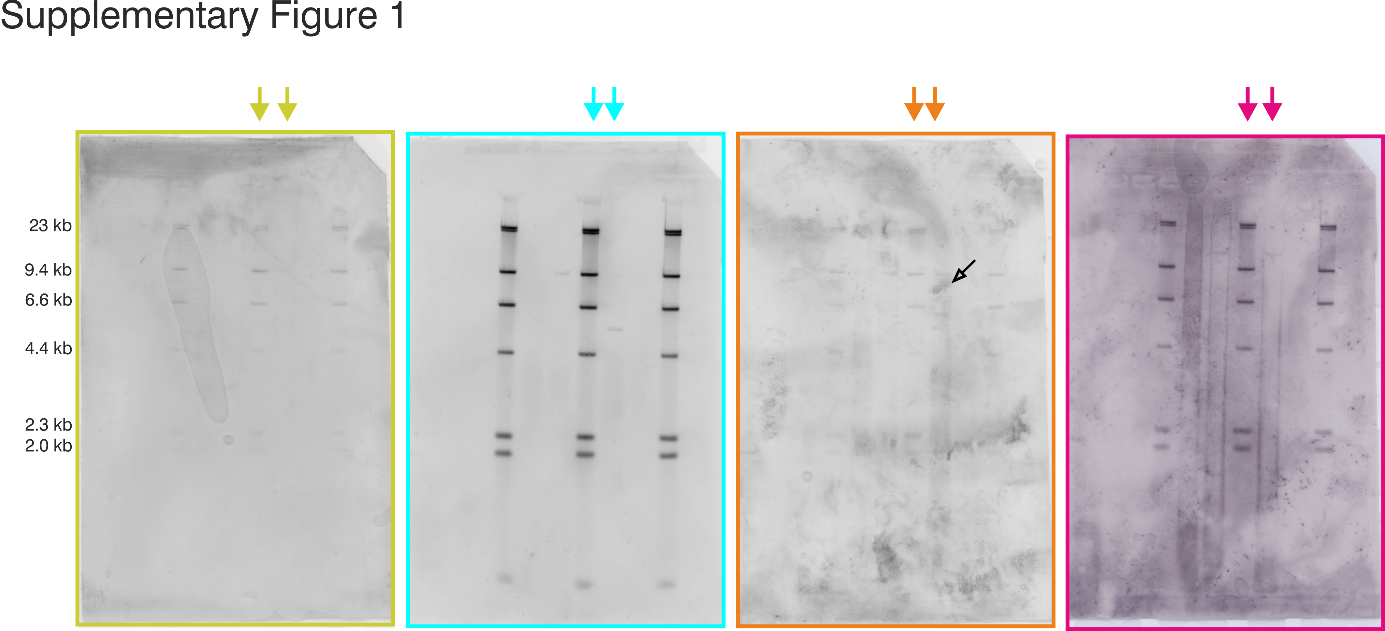
Supplementary Figure 1** Original Southern Blots used for Figure 3 C. Four different probes were used for Southern Blot analyses. Probe 1 (green), specific for the 5´-end: WT band 9.8 kb, transgenic allele 3 kb; probe 2 (blue), covering parts of exon-IV transgene: WT band 9.8 kb, transgenic allele 5.3 kb; probe 3 (orange), covering parts of the exon-VI transgene: WT band 9.8 kb, transgenic allele 6.3 kb; probe 4 (pink), specific for the 3´-end: WT and transgenic allele 12.7 kb. Colored arrows indicate lanes used in Figure 3C. All lanes used for Figure 3C are derived from the same ES cell clone. Southern Blots were inverted and the contrast was increased for all pixels similarly to increase visibility of the band. For probe 3 an unspecific spot was darkened between the 9.8 and 6.3 kb bands, otherwise the bands would not have been clearly visible (black open arrow).


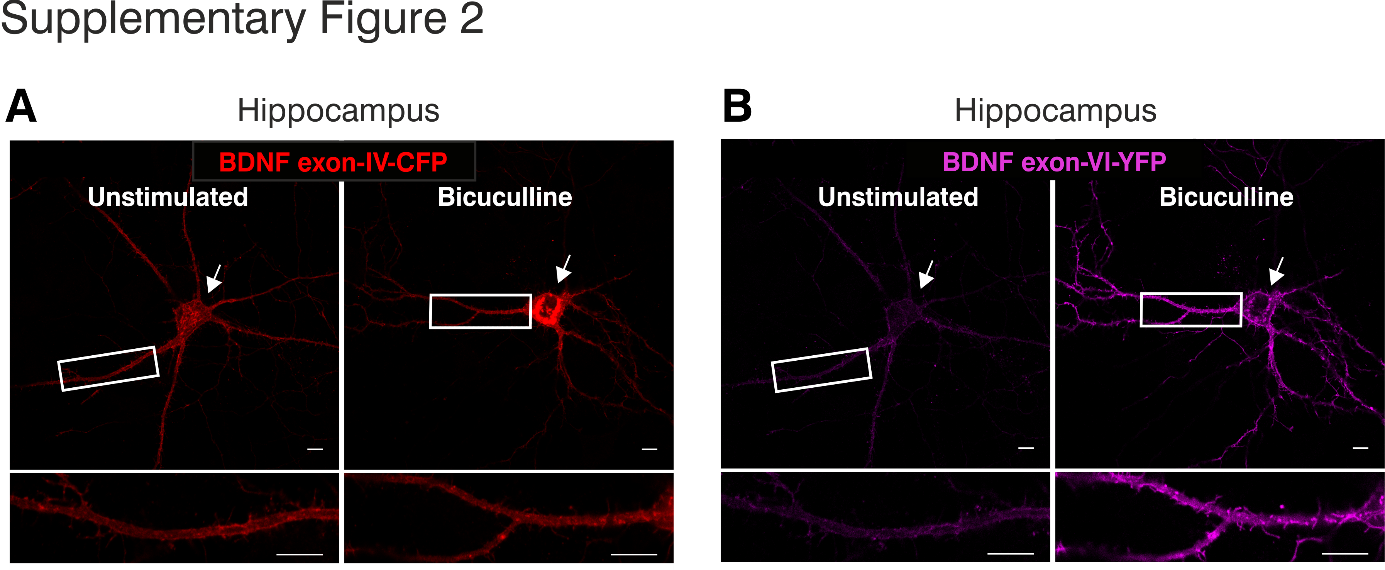


**Supplementary Figure 2** Transfected rat primary hippocampal neurons (embryonic day E18) without (left panel) and with bicuculline (right panel) stimulation. Staining against HA (coupled to *Bdnf* exon-IV-CFP) (**A**) and cMyc (coupled to *Bdnf* exon-VI-YFP) (**B**) was performed with specific antibodies against the HA and c-Myc tags, respectively. Lower panels show higher magnification of area in solid frame. (**A**) Increased expression of *Bdnf* exon-IV-CFP detected preferentially in cell bodies after stimulation with bicuculline (right panel, arrow). (**B**) Increased expression of *Bdnf* exon-VI-YFP after treatment with bicuculline predominantly detected in proximal and distal regions of the neurites (right panel, solid frame). Scale bars: 10 µm**.**

**
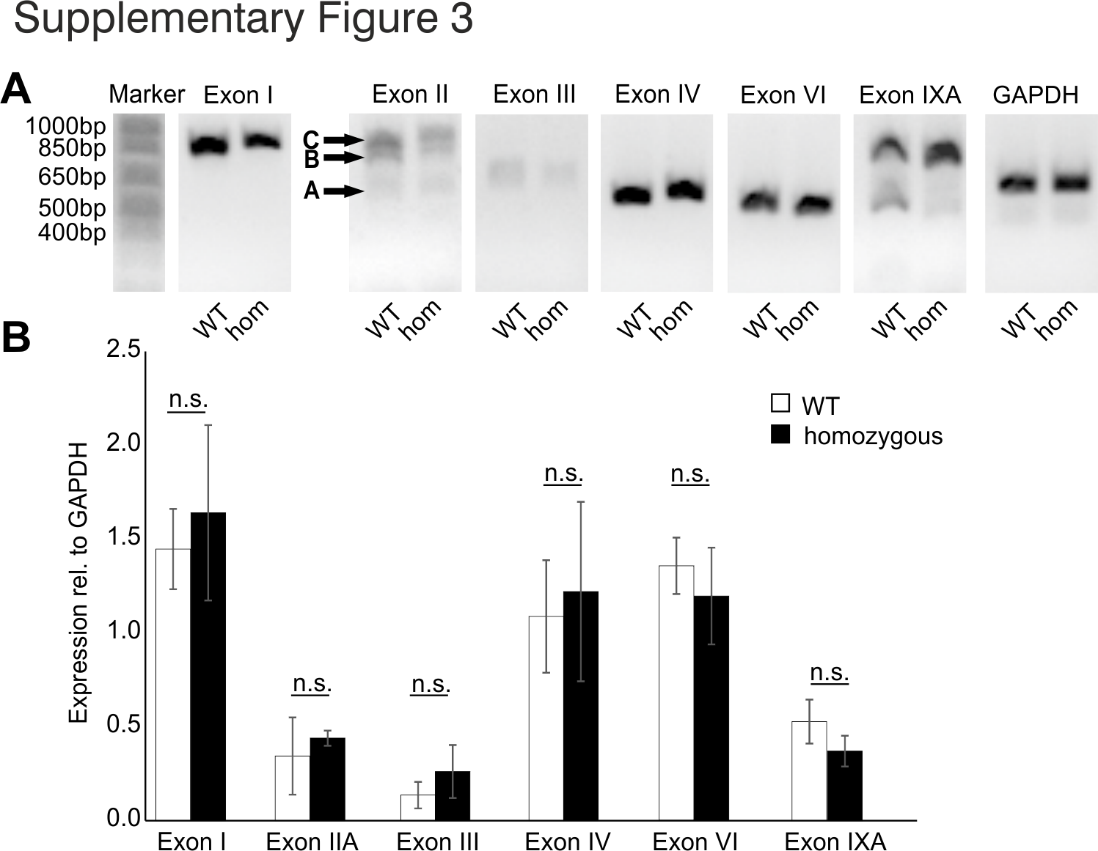
**

**Supplementary Figure 3** Analysis of mRNA expression of the untranslated *Bdnf* exons I, II, III, IV, VI, IXA by RT-PCR in the hippocampus of BLEV mice for wildtype (WT) and homozygous (hom) animals. (**A**) Representative gel picture for *Bdnf* exon I, II, III, IV, VI and IXA. The previously described different transcript variants of *Bdnf* exon II (A, B, C) (Aid et al., 2007) are indicated by arrows. Due to the very similar size of the transcript variants B and C of exon II a separate quantification was not possible. Housekeeping gene: GAPDH. For original picture see Supplementary Figure 6D**.** (**B**) Quantitative analyses of the expression of *Bdnf* exon I, IIA, III, IV, VI, and IXA did not reveal any significant differences between WT and homozygous BLEV mice. Data represented as mean ± SEM (2-sided Student´s *t*-test with α *=*0.05, for details see Supplementary Table 2).

**
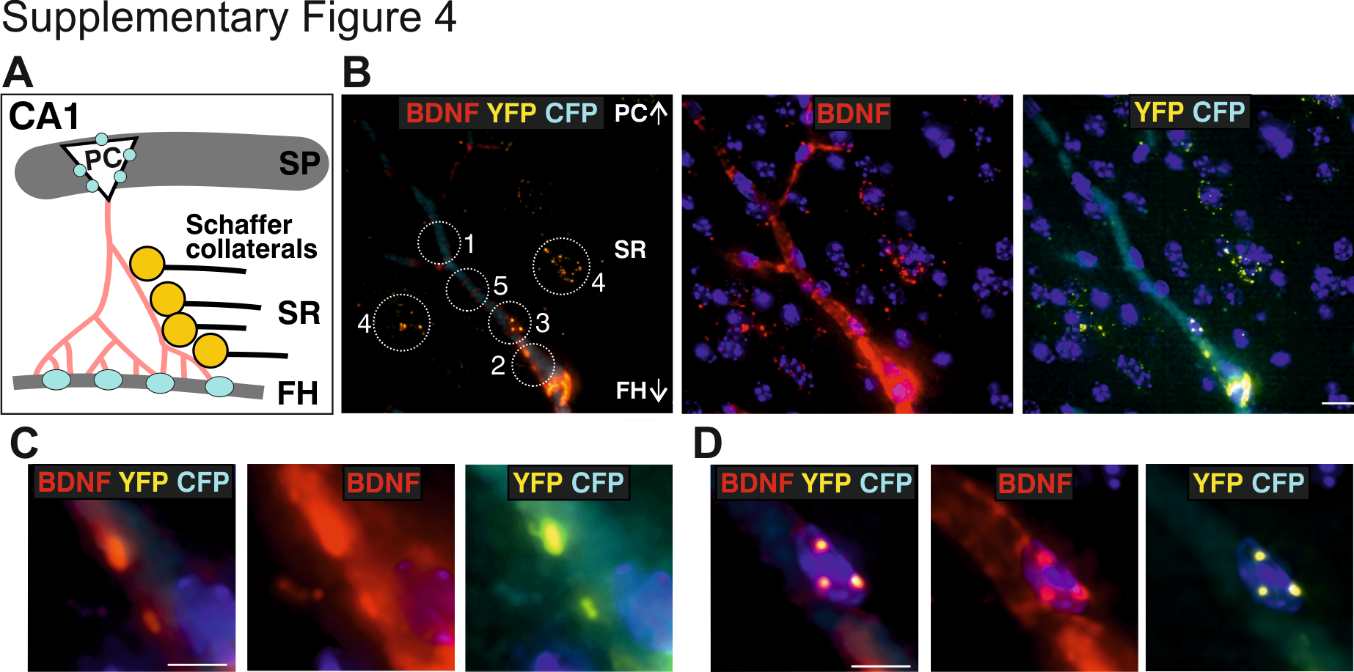
**

**Supplementary Figure 4** Triple images of BDNF IR (red), YFP and CFP (**A**) Schematic overview of the CA1 region containing the stratum pyramidale (SP), the stratum radiatum (SR) and the fissura hippocampalis (FH). (**B**) Merged image of Figure 5B. Left picture: BDNF-IR in 5 different characteristic regions at the level of the SR co-localized with *Bdnf* exon-VI-YFP and *Bdnf* exon-IV-CFP. Middle picture: BDNF-IR (red). Right picture: Co-localization of *Bdnf* exon-VI-YFP and *Bdnf* exon-IV-CFP. Nuclei were stained with DAPI (blue). Scale bars: 100 μm. (**C**) Merged image of Figure 5E. Left picture: BDNF-IR co-localized with *Bdnf* exon-VI-YFP close to a *Bdnf* exon-IV-CFP positive capillary in the FH. Middle picture: BDNF-IR (red). Right picture: Co-localization of *Bdnf* exon-VI-YFP and *Bdnf* exon-IV-CFP. Nuclei were stained with DAPI (blue). Scale bar: 10 μm (**D**) Merged image of Figure 5H. Left picture: BDNF-IR co-localized with *Bdnf* exon-VI-YFP-positive puncta on *Bdnf* exon-IV-CFP-positive capillaries. Middle picture: BDNF-IR (red). Right picture: Co-localization of *Bdnf* exon-VI-YFP and *Bdnf* exon-IV-CFP. Nuclei were stained with DAPI (blue). Scale bar: 10 μm.


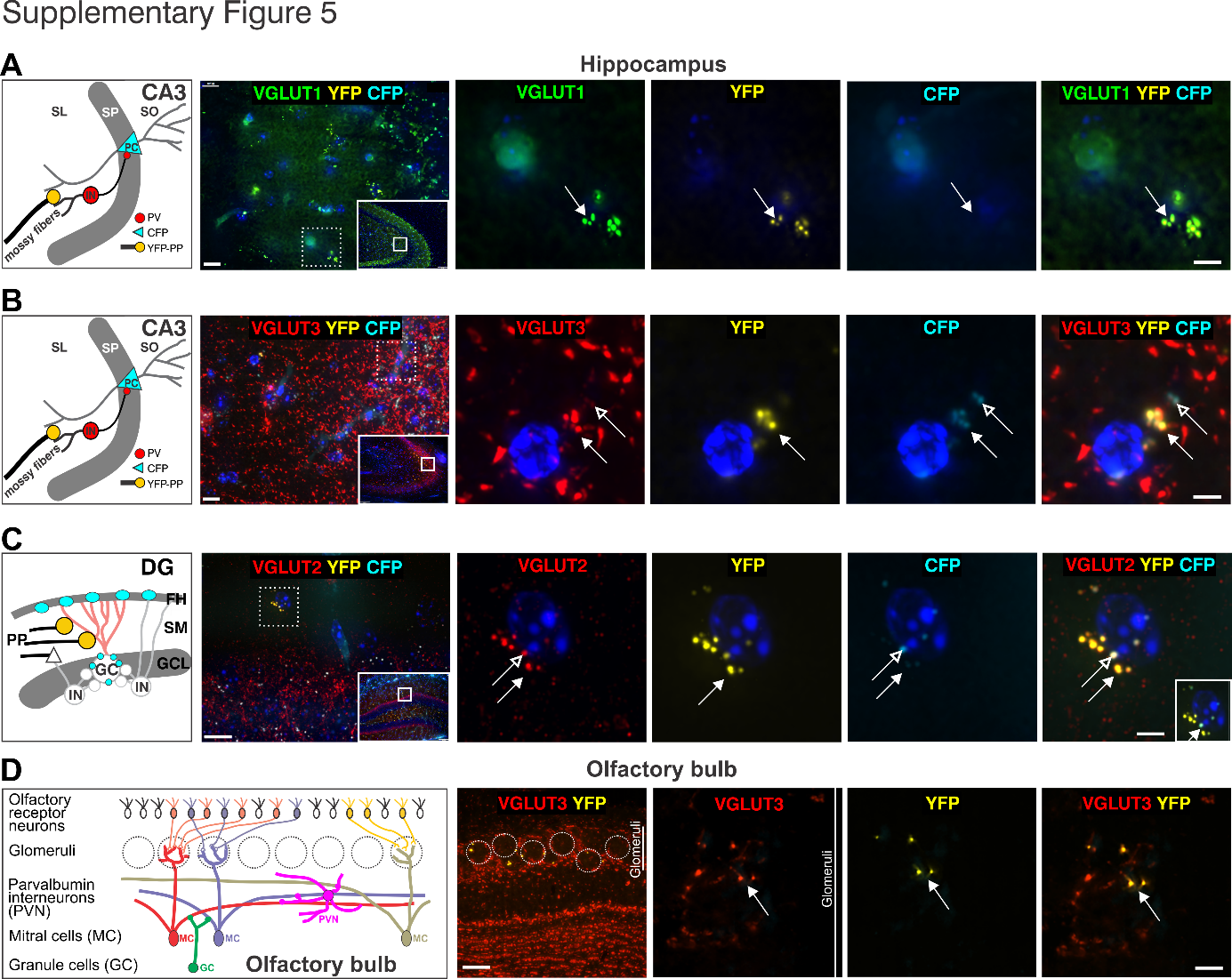


**Supplementary Figure 5** Immunostaining of the presynaptic marker proteins VGLUT1, VGLUT2 and VGLUT3. (**A**) Schematic overview of the CA3 region containing the stratum lucidum (SL), stratum pyramidale (SP), the stratum oriens (SO), parvalbumin (PV) positive interneurons (IN) and pyramidal cells (PC). Second panel: VGLUT1 IR in green co-localized with *Bdnf* exon-VI-YFP and *Bdnf* exon-IV-CFP within the CA3 region. Inset: lower magnification of the same area (solid frame). Scale bar: 100 μm. Right panels: higher magnification depicting single channels of VGLUT1 (green), YFP and CFP demonstrate co-localized VGLUT1 and YFP positive dots (arrows). Scale bar: 10 μm. Nuclei were stained with DAPI (blue) (**B**) Schematic overview of the CA3 region. Second panel: VGLUT3 IR in red co-localized with *Bdnf* exon-VI-YFP and *Bdnf* exon-IV-CFP. Inset lower magnification of the area (solid frame). Scale bar: 100 μm. Right panels: higher magnification depicting single channels of VGLUT3 (red), YFP and CFP show co-localization of VGLUT3 and YFP (arrows). Some few dots show a co-localization of VGLUT3 (red) and CFP (open arrows). Scale bar: 10 μm.. Nuclei were stained with DAPI (blue) **(C)** Schematic overview of the dentate gyrus (DG) region containing the fissura hippocampalis (FH), stratum moleculare (SM), the granular cell layer (GCL), granular cells (GC), interneurons (IN) and the perforant path (PP). Second panel: VGLUT2 IR (red) co-localized with *Bdnf* exon-VI-YFP and *Bdnf* exon-IV-CFP. Inset: lower magnification of the same area (solid frame). Scale bar: 100 μm. Right panels: higher magnification depicting single channels of VGLUT2, YFP and CFP, which demonstrate a co-localization of VGLUT2 (red) and YFP (arrows). Some few dots show a co-localization of VGLUT2 (red), YFP and CFP (open arrows). Inset in the right panel shows Scale bar: 10 μm. Nuclei were stained with DAPI (blue). (**D**) Olfactory bulb. Left panel: Schematic overview of the olfactory bulb containing the olfactory receptor neurons, the glomeruli, parvalbumin–positive interneurons (PVN), the mitral cells (MC), and the granule cells (GC). Second panel: VGLUT3 IR (red) in the olfactory bulb co-localized with *Bdnf* exon-VI-YFP. Scale bar: 100 μm. Right panels: higher magnification of the glomeruli. A clear co-localization of VGLUT3 IR (red) and *Bdnf* exon-VI-YFP was observed within the glomeruli. Scale bar: 10 μm.


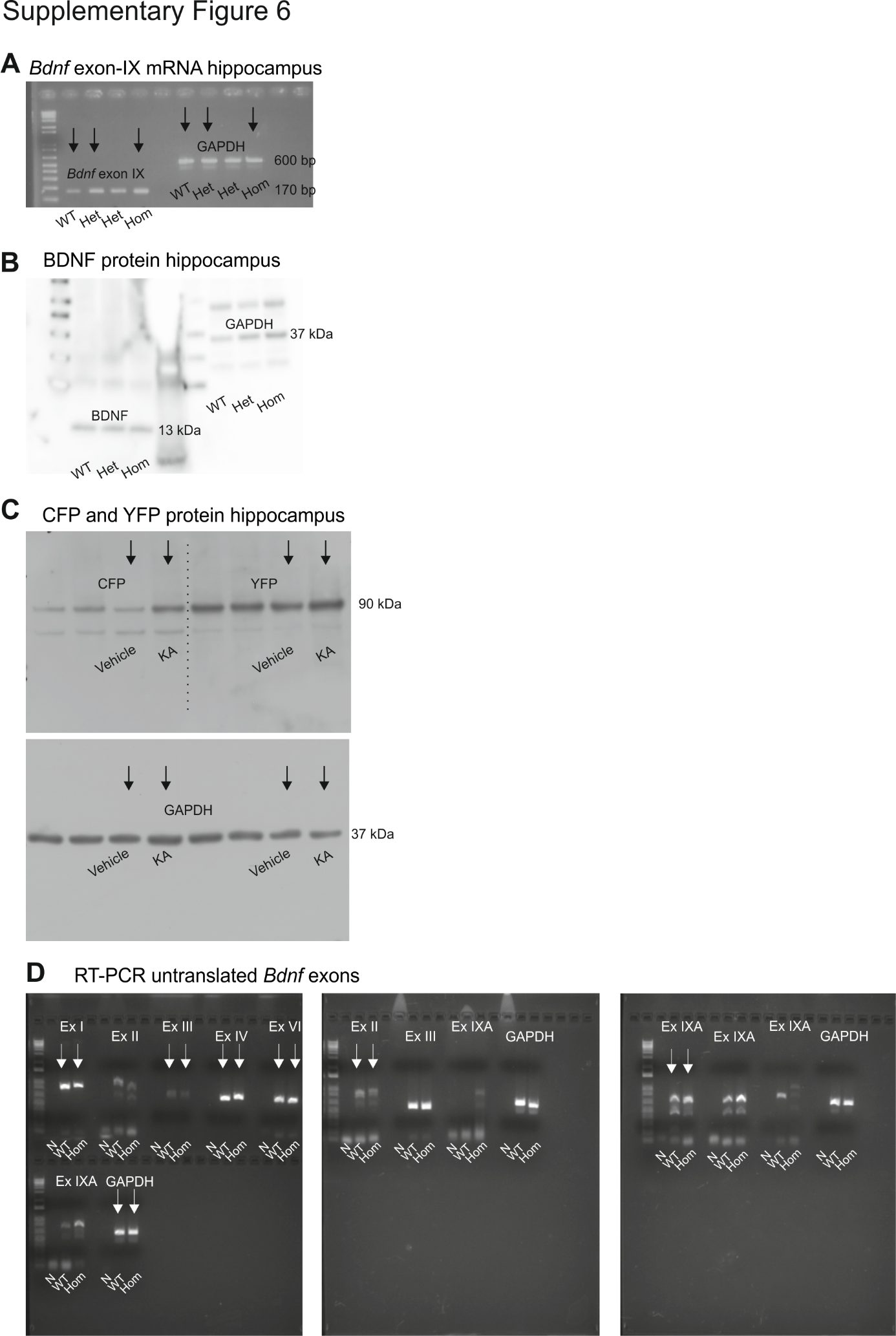


**Supplementary Figure 6** Original PCR and Western Blots for Figures 4C/D and 7A. (**A**) Picture from the original PCR gel used for Figure 4C. Black arrows indicate bands used for the figure. As two heterozygous animals were used for PCR, one is left out. (**B**) Original Western blot used for Figure 4D. Contrast was increased for better visibility. For quantification the unmodified image was used. (**C**) Original Western blot used for Figure 7A. Black arrows indicate bands used for the figure. As other probes were run on the same blot relevant bands were cut. Contrast was increased for better visibility. For quantification the unmodified image was used. (**D**) Original PCR gel used for Supplementary Figure 2. Arrows indicate the used lanes. For quantification the unmodified images were used.

**Supplementary Table 1** Antibody information for immunohistochemistry and Western blot

| Primary Antibodies |  |  |  |  |  |  |
| --- | --- | --- | --- | --- | --- | --- |
| Immunohistochemistry | Antibody | Protein name | Product number | Source | Dilution | Protein size |
| Brain sections | Mouse anti-BDNF | Brain-derived neurotrophic factor | BDNF #9-b | Developmental Studies Hybridoma Bank University of Iowa | 1:50 |  |
|  | Rabbit anti-parvalbumin | Parvalbumin | ab11427 | Abcam | 1:2,000 |  |
|  | Mouse anti-desmin | Desmin | ab8976 | Abcam | 1:100 |  |
|  | Rabbit anti-Arc | Activity regulated cytoskeletal protein | 156 003 | Synaptic Systems | 1:500 |  |
|  | Rabbit anti-IBA-1 | Ionized calcium-binding adaptor molecule 1 | 016-20001 | Wako Chemicals | 1:500 |  |
|  | Rabbit anti-GFAP | Glial fibrillary acidic protein | Z 0334 | Dako | 1:2,000 |  |
|  | Rabbit anti-p75NGFR | p75 neurotrophin receptor | AB1554 | Millipore | 1:100 |  |
|  | Rabbit anti-b1-GC | b1-subunit of the nitric oxide-sensitive guanylate cyclase |  | Müllerhausen et al. 2004 | 1:200 |  |
|  | Guinea pig  anti-vGLUT1 | Vesicular glutamate transporter 1 | 135304 | Synaptic System | 1:1,500 |  |
|  | Rabbit  anti-vGLUT2 | Vesicular glutamate transporter 2 | 135403 | Synaptic System | 1:500 |  |
|  | Rabbit  anti-vGLUT1 | Vesicular glutamate transporter 3 | 135203 | Synaptic System | 1:1,000 |  |
| Cell culture | Mouse anti-Myc | Myc-tagged protein | 2276 | Cell Signaling | 1:500 |  |
|  | Rabbit  Anti-HA | HA-tagged protein | AB9110 | Abcam | 1:500 |  |
| Western blot | Rabbit anti-RCFP | Reef coral fluorescent protein pan antibody | 632475 | Clontech | 1:1,000 | 30-35 kDa |
|  | Rabbit anti-BDNF | Brain-derived neurotrophic factor | sc-546 | Santa Cruz Biotechnology Inc. | 1:400 | 15 kDa |
|  | Mouse anti-GAPDH | Glyceraldehyde 3-phosphate dehydrogenase | ab8245 | Abcam | 1:10,000 | 40 kDa |
| Secondary antibodies |  |  |  |  |  |  |
| Immunohistochemistry |  |  |  |  |  |  |
| Brain sections |  |  |  |  |  |  |
|  | Antibody | Protein name | Product number | Source | Dilution | Protein size |
|  | Cy3-conjugated goat anti-mouse antibody |  | 115-1665-062 | Jackson ImmunoResearch Laboratories | 1:3,000 |  |
|  | AlexaFluor 488 goat anti-guinea pig |  | A-11073 | Molecular Probes | 1:500 |  |
| Cell culture | AlexaFluor 568 conjugated goat anti-rabbit |  | A-11011 | Invitrogen | 1:500 |  |
|  | AlexaFluor 647 conjugated goat anti-mouse |  | A-21235 | Invitrogen | 1:500 |  |
| Western blot | Cy3-conjugated goat anti-rabbit antibody |  | 111-166-003 | Jackson ImmunoResearch Laboratories | 1:1,500 |  |
|  | Alexa488-conjugated anti-mouse antibody |  | A11001 | Molecular Probes, MoBiTec | 1:500 |  |
|  | Alexa488-conjugated anti-guinea pig antibody |  | A11073 | Molecular Probes, MoBiTec | 1:500 |  |
|  | ECL anti-mouse IgG HRP linked |  | NA 931-100µl | GE Healthcare UK Limited | 1:2,500 |  |
|  | ECL anti-rabbit IgG HRP linked |  | NA 934-100µl | GE Healthcare UK Limited | 1:2,500 |  |

**Supplementary Table 2** Statistical information

|  |  | **Statistical test** | **Test value** | **Degrees of freedom** | ***p*-value** | **Post-hoc test with *p*-value** |  | **n-number** |
| --- | --- | --- | --- | --- | --- | --- | --- | --- |
| Fig. 1E |  | 1-way ANOVA |  |  | p = 0.0002 | post-test 2-sided Student´s t-test |  | done in duplicate for 4 independent experiments |
|  |  |  | F (3, 36) = 8.35 |  |  | CFP | p = 0.008 |  |
|  |  |  |  |  |  | YFP | p = 0.003 |  |
| Fig. 4B |  |  |  |  |  |  |  | WT n = 9  Het n = 11  Hom n = 11 animals |
| Fig. 4C | PCR (mRNA) | 1-way ANOVA | F(2,9) = 0.15 |  | p = 0.86 |  |  | n = 3 animals / genotype |
| Fig. 4D | Westen blot (Protein) | 1-way ANOVA | F(2,12) = 0.20 |  | p = 0.82 |  |  | n = 4 animals / genotype |
| Fig. 4E | click-ABR | 1-way ANOVA | F(2, 26) = 1.988 |  | p = 0.16 |  |  | WT n = 20/10 ears/animals het n = 30/15  ears/ animals hom n = 10/5  ears/animals |
|  | f-ABR | 2-way ANOVA | F(18,  2665) = 0.50 | | p = 0.96 |  |  |  |
| Fig. 7A | Western blot  (CFP) | 1-sided Student’s *t*-test | t = 6.25 | DF = 4 | p = 0.002 |  |  | n = 3 animals / group |
|  | Western blot  (YFP) | 1-sided Student’s *t*-test | t = 1.99 | DF = 6 | p = 0.04 |  |  | n = 4 animals / group |
| Fig. 7D | Integrated density (YFP) | 1-sided Student’s *t*-test | t = 4.14 | DF = 3 | p = 0.0128 | |  | n = 3 animals / group |
| Fig. 7F | Integrated density (CFP) | 1-sided Student’s *t*-test | t = 2.32 | DF = 4 | p = 0.0405 | |  | n = 2-3 animals / group |
| Supp. Fig. 2B | PCR (mRNA) | 2-sided Student’s *t*-test | Exon I t = 0.7327 Exon IIA t = 0.3913 Exon III t = 1.007 Exon IV t = 0.3009 Exon VI t = 0.5474 Exon IXA t = 0.9908 | DF = 6 DF = 6 DF = 6 DF = 6 DF = 6 DF = 6 | p = 0.4914 p = 0.7091 p = 0.3526 p = 0.7737 p = 0.6132 p = 0.3560 |  |  | n = 3 animals / genotype; 4-5 replicates / animal |
